# Basophil Depletion Alters Host Immunity, Intestinal Permeability, and Mammalian Host-to-Mosquito Transmission in Malaria

**DOI:** 10.4049/immunohorizons.2200055

**Published:** 2022-08-15

**Authors:** Erinn L. Donnelly, Nora Céspedes, Gretchen Hansten, Delaney Wagers, Anna M. Briggs, Casey Lowder, Joseph Schauer, Sarah M. Garrison, Lori Haapanen, Judy Van de Water, Shirley Luckhart

**Affiliations:** * Department of Biological Sciences, University of Idaho, Moscow, ID; † Department of Entomology, Plant Pathology and Nematology, University of Idaho, Moscow, ID; ‡ Division of Rheumatology, Allergy and Clinical Immunology, University of California, Davis, Davis, CA

## Abstract

Malaria-induced bacteremia has been shown to result from intestinal mast cell (MC) activation. The appearance of MCs in the ileum and increased intestinal permeability to enteric bacteria are preceded by an early Th2-biased host immune response to infection, characterized by the appearance of IL-4, IL-10, mast cell protease (Mcpt)1 and Mcpt4, and increased circulating basophils and eosinophils. Given the functional similarities of basophils and MCs in the context of allergic inflammation and the capacity of basophils to produce large amounts of IL-4, we sought to define the role of basophils in increased intestinal permeability, in MC influx, and in the development of bacteremia in the context of malaria. Upon infection with nonlethal *Plasmodium yoelii yoelii* 17XNL, Basoph8 × ROSA-DTα mice or baso (−) mice that lack basophils exhibited increased intestinal permeability and increased ileal MC numbers, without any increase in bacterial 16S ribosomal DNA copy numbers in the blood, relative to baso (+) mice. Analysis of cytokines, chemokines, and MC-associated factors in the ileum revealed significantly increased TNF-α and IL-13 at day 6 postinfection in baso (−) mice compared with baso (+) mice. Moreover, network analysis of significantly correlated host immune factors revealed profound differences between baso (−) and baso (+) mice following infection in both systemic and ileal responses to parasites and translocated bacteria. Finally, basophil depletion was associated with significantly increased gametocytemia and parasite transmission to *Anopheles* mosquitoes, suggesting that basophils play a previously undescribed role in controlling gametocytemia and, in turn, mammalian host-to-mosquito parasite transmission.

## INTRODUCTION

Malaria remains one of the largest public health challenges, with an estimated 241 million new infections and 627,000 deaths in 2020 (1). Concomitant bacteremia with malaria has been reported in both children (2, 3) and adults (4, 5). Metaanalysis of 25 studies on this topic estimated the prevalence of bacteremia in children with severe falciparum malaria to be 6.4% (3). The prevalence of bacteremia in adults is less well studied and the estimates vary widely. For example, studies in Myanmar reported 13–15% prevalence of bacteremia in adults with falciparum malaria (4, 5), whereas a study from Vietnam reported 1% prevalence (6). Nevertheless, both pediatric and adult patients with malaria and bacteremia have more severe disease and higher case fatality rates than do patients with malaria alone (3-6).

Previous studies in mice have demonstrated that the influx of mast cells (MCs), or mastocytosis, in the intestine is necessary for the development of bacteremia (7, 8). Furthermore, supplementation with l-arginine and l-citrulline reduced malaria-induced intestinal mastocytosis and significantly decreased bacteremia (7). Additionally, MC-deficient mice infected with nonlethal *Plasmodium yoelii yoelii* 17XNL showed reduced intestinal permeability and bacteremia relative to nondeficient controls (8). Treatment with antihistamine partially protected against increased intestinal permeability, epithelial damage, and bacteremia (8), supporting the involvement of MCs and/or their products in malaria-induced gut barrier disruption.

Céspedes et al. (9) presented a detailed timeline of *P. y. yoelii* 17XNL infection, noting an early type 2–skewed host immune response to infection (9). Specifically, the anti-inflammatory cytokines IL-4 and IL-10, mast cell protease (Mcpt)4, eosinophils, and basophils were significantly increased in circulation at 4 d postinfection (p.i.). Basophils are of particular interest as a potential driver of intestinal mastocytosis, as they are strongly activated by parasite translationally controlled tumor protein (TCTP) (10) and are known to release factors that activate MCs (11). In particular, activated basophils release IL-4, IL-13, and histamine (12) and are known to be a source of IL-4 in parasitic helminth infections (13). In the context of malaria, however, the role of basophils in disease pathogenesis is not well understood. Infection with *Plasmodium chabaudi chabaudi* AS induces an increase in basophil numbers in the spleen around the time of peak parasitemia, and stimulation with IL-3 can cause these cells to produce IL-4 (14). Bakocevic et al. (15) showed that basophils are activated rapidly in *P. yoelii* 17X-infected mice, with significant movement of these cells into liver and spleen by day 3 p.i., the first day of detectable peripheral parasitemia (15). Infection of mcpt8DTR mice treated with diphtheria toxin (DT) prior to infection to deplete basophils, however, was associated with only a small decrease in *P. yoelii* parasitemia compared with controls without DT treatment (15). Another study utilizing *Plasmodium berghei* ANKA as a model of cerebral malaria found that depletion of neither basophils nor MCs altered disease progression and survival outcomes (16).

To address some of these gaps in knowledge regarding the function of basophils in malaria, and to directly test the hypothesis that basophils control malaria-induced intestinal permeability and bacteremia, we infected basophil-deficient Basoph8 × ROSA-DTα mice with *P. y. yoelii* 17XNL and compared them against nondeficient Basoph8 and ROSA-DTα controls. Our data revealed that basophil depletion is associated with increased intestinal permeability and mastocytosis in malaria and, quite unexpectedly, with increased circulating gametocytemia and malaria parasite transmission to the mosquito host, *Anopheles stephensi*.

## MATERIALS AND METHODS

### Mice

Baso (−) mice were generated by crossing Basoph8 mice (stock no. 017578) with ROSA-DTα mice (stock no. 009669) as described (17). Both lines were purchased from The Jackson Laboratory. A total of 79 Basoph8 × ROSA-DTα mice (hereafter referred to as baso (−) mice) were used as experimental animals, and sex- and aged-matched littermate ROSA-DTα (*n* = 61) or Basoph8 mice (*n* = 27) were used as baso (+) controls. Experimental procedures were conducted on both male mice (*n* = 62) and female mice (*n* = 105) at 6–8 wk of age. Mice were housed in ventilated microisolator caging and provided food and water ad libitum. All procedures were approved by the Institutional Animal Care and Use Committee of the University of Idaho (protocol no. IACUC-2020-10, approved March 30, 2020).

### Mouse infection and monitoring

A total of 79 baso (−) mice and 88 baso (+) controls (61 ROSA-DTα and 27 Basoph8 mice) were used across four replicate studies of parasite infection. Mice were infected by i.p. injection of 150 μl of *P. y. yoelii* 17XNL–infected RBCs or iRBCs (1 × 10^6^ parasites) (*n* = 133) or USP saline (*n* = 34) at day 0. Mice were sacrificed at 3, 4, 6, 8, and 10 d p.i. for blood and/or tissue collection. Starting at 2 d p.i., daily parasitemia levels were recorded from thin blood smears stained with Giemsa. To calculate peripheral parasitemia, the numbers of RBCs infected with asexual parasites or gametocytes were counted and divided by the total number of RBCs counted in five fields viewed at ×1000 magnification on a light microscope. To calculate gametocytemia at 3 d p.i., the numbers of RBCs infected with gametocytes were counted and divided by the total number of RBCs counted in 25 fields viewed at ×1000 magnification. Mice were monitored daily for weight loss and reduced activity to determine humane endpoints. Blood samples were collected by cardiac puncture for determination of bacterial 16S ribosomal DNA copies. Plasma samples were collected for quantitation of IgE, cytokines, chemokines, Mcpt4, and Mcpt1. These samples were stored at −80°C until analysis. Ileum tissue was collected and divided into a snap-frozen portion for cytokine and chemokine analysis and into a formalin-fixed portion for histology.

### In vivo intestinal permeability

In one replicate of 40 female mice (17 baso (−) infected mice, 3 baso (−) uninfected mice, 17 baso (+) infected mice, and 3 baso (+) uninfected mice), intestinal permeability was quantified as described (9). Briefly, mice were fasted for 4h before oral gavage of 4-kDa FITC dextran (MilliporeSigma, Burlington, MA) in sterile saline at a dose of 50 mg/100 g body weight. After 3 h, blood was collected and plasma was separated. Plasma was diluted 1:2 v/v with PBS, and fluorescence was measured using a microplate reader (Molecular Devices, San Jose, CA) at excitation/emission of 490/520 nm.

### Ileum histochemistry and MC staining

Formalin-fixed ileum samples were embedded in paraffin tissue blocks and 5-μm sections were cut, deparaffinized in xylene, rehydrated in graded solutions of alcohol, and subjected to enzyme histochemical staining to identify naphthol AS-D chloroacetate esterase (NASDCE) activity (91C-1KT; Sigma-Aldrich, St. Louis, MO), allowing for the visualization of chymases in MC secretory granules (18). For each mouse examined (*n* = 78; 37 baso (−), 41 ROSA-DTα), MCs were enumerated in 30–50 high-power fields (HPFs).

### ELISAs

Levels of plasma IgE (eBioscience, San Diego, CA; Thermo Fisher Scientific, Waltham, MA), Mcpt4 (Aviva Systems Biology, San Diego, CA), Mcpt1 (eBioscience), and histamine (Enzo Life Sciences, Farmingdale, NY) were determined in plasma samples using commercial ELISAs according to the manufacturers’ instructions and a microplate reader (BMG Labtech, Cary, NC).

### Extraction of DNA from blood and 16S quantitative PCR

In all mice, except those used in flow cytometry or transmission studies (*n* = 153), bacterial 16S copies were determined using quantitative PCR primers (forward, 5′-ACTCCTACGGGAGGCAGCAGT-3′, reverse, 5′-ATTACCGCGGCTGCTGGC-3′) and conditions previously described (9). Briefly, DNA was isolated from whole blood using a DNeasy Blood & Tissue kit (Qiagen, Germantown, MD) according to the manufacturer’s protocol. DNA was diluted to 4 ng/μl and samples were assayed in triplicate using Maxima SYBR Green/ROX quantitative PCR master mix (2×) (Thermo Fisher Scientific), with 16S forward and reverse primers at a final concentration of 0.4 μM. Copies were quantified using a standard curve of 16S bacterial DNA plasmid DNA. Data were analyzed using QuantStudio 6 Flex (Applied Biosystems).

### Cytokines and chemokines in plasma and ileum samples

Concentrations of plasma cytokines and chemokines (IL-1α, IL-1β, IL-2, IL-3, IL-4, IL-5, IL-6, IL-9, IL-10, IL-12p40, IL-12p70, IL-13, IL-17, eotaxin, G-CSF, GM-CSF, IFN-γ, KC, MCP-1, MIP-1α, MIP-1β, RANTES, and TNF-α) were determined in all mice (*n* = 163, excluding *n* = 4 used in flow cytometry studies) as previously described (9). Concentrations of cytokines and chemokines (identical to those measured in plasma, with the addition of IL-33) in 2 cm of ileum tissue were determined in the 153 mice not used for transmission studies (*n* = 10) or flow cytometry studies (*n* = 4). Ileum tissue was collected at necropsy, weighed, snap-frozen, and stored at −80° C until processing for protein isolation using the Bio-Plex cell lysis kit (Bio-Rad, Hercules, CA), as described (19). Briefly, tissue samples were washed with lysis solution and lysed by probe sonication (Branson SFX150, Branson, Danbury, CT) followed by water bath sonication for 3 min and centrifugation at 4500 × *g* for 4 min at 4°C. After measuring protein concentration using a Pierce bicinchoninic acid assay (Thermo Fisher Scientific), cytokine and chemokine profiles were determined for each ileum sample (in duplicate) by a Bio-Plex Pro Luminex assay.

### Transmission studies

To assess the effect of basophil depletion on parasite transmission, *A. stephensi* (Indian strain, reared as described in Ref. 20), were fed on 8- to 10-wk-old male baso (−) mice (*n* = 5) or baso (+) (Basoph8) mice (*n* = 5) at 3 d p.i. A drop of blood was used to make wet prep slides to check for exflagellation of male gametocytes. Exflagellation events were counted as events per field on ×200 magnification. A total of four fields were examined per mouse, and mice with similar numbers of average exflagellation events (11–15 events/field) were chosen to infect mosquitoes. Each mouse was anesthetized with ketamine (50 mg/kg) and xylazine (5 mg/kg) in sterile saline and placed on top of a carton containing ~60 female mosquitoes (3–5 d old). Mosquitoes were allowed to feed for 15 min. After feeding was complete, mice were euthanized via CO_2_ asphyxiation followed by cervical dislocation. At the time of euthanasia, blood samples were collected for plasma preparation and cytokine analysis. Non–blood-fed mosquitoes were removed from the cartons. At 10 d after feeding, 25–35 mosquitoes per carton were dissected to quantify numbers of oocysts per midgut (infection intensity) and numbers of infected mosquitoes (the presence of at least one midgut oocyst or infection prevalence). Midguts were dissected and stained with mercurochrome and oocysts per midgut were counted on a light microscope.

### Flow cytometry

To confirm depletion of basophils (Supplemental Fig. 1), spleens were collected from male baso (−) mice (*n* = 2) and baso (+) mice (Basoph8, *n* = 2) at 4 d p.i. for flow cytometry. Spleens were passed through a 70-μm nylon cell strainer into 1% BSA in PBS. After RBC lysis (10× RBC lysis buffer multispecies, eBioscience), cells were pelleted at 500 × *g* for 5 min, washed once with 1% BSA, and counted. A total of 10^7^ cells per spleen were incubated with anti-mouse CD49b (PerCP) (eBioscience) and anti-mouse FCεRlα (allophycocyanin) (eBioscience) at the manufacturers’ recommended concentrations for 1 h at room temperature, protected from light, and then stained with DAPI (Invitrogen). Cells were then fixed for 20 min in 1% formalin, washed, and counted using a CytoFLEX flow cytometer (Beckman Coulter, Brea, CA). Live granulocytes were selected based on forward/side scatter and DAPI staining, and basophils within this population were defined as expressing both CD49b and FCεR1α (15, 21). Data were analyzed with CytExpert software (Beckman Coulter).

### Statistical analysis

Parasitemia, intestinal permeability, bacterial 16S DNA copies per microliter of blood, Mcpt1, Mcpt4, IgE, MCs per HPF, and cytokine/chemokine concentrations were analyzed by the ROUT method (maximum false discovery rate *Q* = 1%) to exclude outliers. Non-normal data were compared among time points using the Kruskal–Wallis test followed by a Dunn’s multiple comparison test of each time point between genotypes. Normally distributed data were analyzed using Brown–Forsythe and Welch ANOVA. Parasitemia, gametocytemia, cytokines/chemokine levels, MC numbers, Mcpt1, Mcpt4, histamine, IgE, and 16S data from male and female mice of the same genotype at the same time point were analyzed by a Kruskal–Wallis test followed by a Dunn’s multiple comparison test (non-normal data) or by Brown–Forsythe and Welch ANOVA (normally distributed data). When no significant differences were observed between the sexes, data were plotted together. When significant differences between the sexes were observed at any time point for a measured parameter, all data for that parameter were plotted separately. Mean oocysts per midgut and gametocytemia were compared using an unpaired *t* test with Welch’s correction. Infection prevalence was analyzed by Fisher’s exact test. A *p* value <0.05 for all analyses was considered significant.

### Network analysis

Correlations among parasitemia, gametocytemia, oocyst numbers for mosquitoes fed on a single mouse, 16S copies, MCs per HPF, IgE, Mcpt1, Mcpt4, histamine, and plasma and ileum cytokines and chemokines were analyzed using a Spearman test at 3, 4, 6, 8, and 10 d p.i. Network analysis was performed with Cytoscape (https://cytoscape.org) version 3.8.2 using significant Spearman correlations (*p* ≤ 0.05). Parasitemia, gametocytemia, oocyst number, and 16S copies were used as main targets, and MCs per HPF, IgE, Mcpt1, and plasma and ileum cytokines and chemokines were used as sources. Only sources with > 1.5-fold change relative to uninfected mice were included in network analyses.

### Ethics statement

All experiments were performed with the approval of the Institutional Animal Care and Use Committee of the University of Idaho (protocol no. IACUC-2020-10, approved March 30, 2020).

## RESULTS

### Basophil depletion did not alter peripheral parasitemia relative to baso (+) controls

**FIGURE 1.**
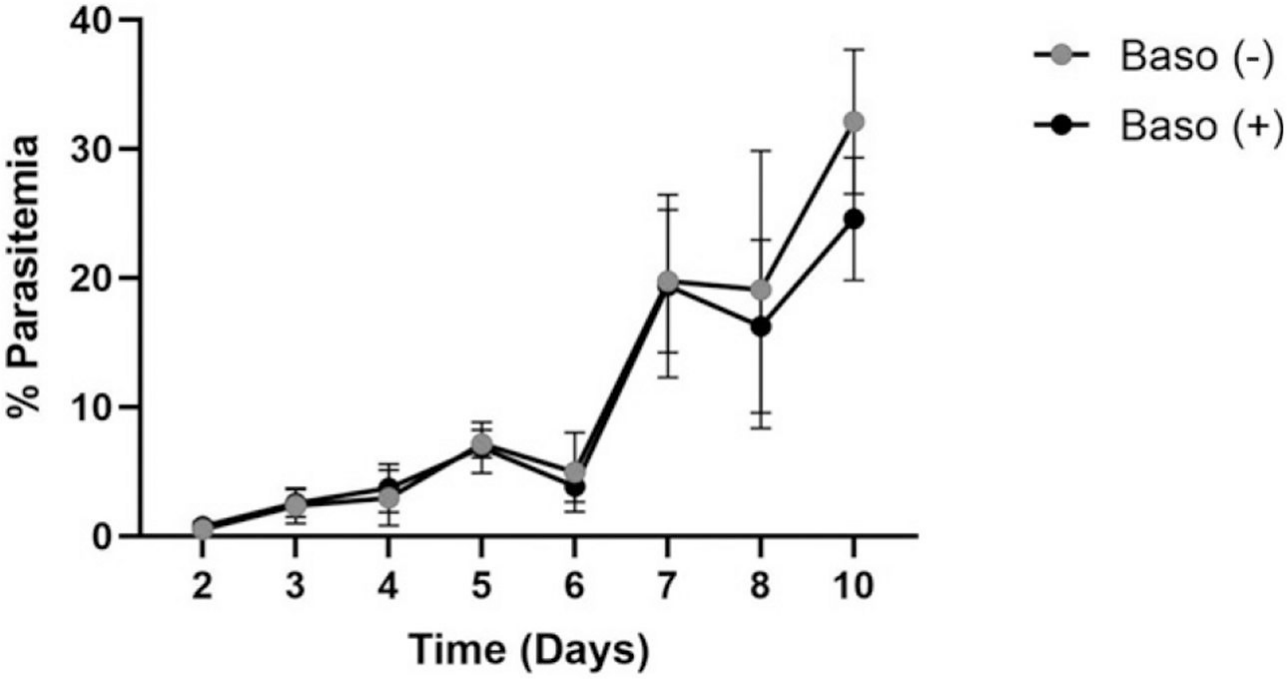
Peripheral parasitemias were similar following *P. y. yoelii* 17XNL infection in basophil-depleted mice and nondepleted mice. Peripheral parasitemia was quantified as the number of RBCs infected with asexual parasites or gametocytes divided by the total number of RBCs counted in five fields viewed at ×1000 magnification on a light microscope. Error bars represent means ± SD. Data were analyzed with a Kruskal–Wallis test followed by a Dunn’s multiple comparison test between genotypes at each time point. A *p* value < 0.05 was considered significant. Baso (–), basophil-depleted mice (Basoph8 + ROSA-DTA, *n* = 62): baso (+) nondepleted mice (Basoph8, *n* = 24 or ROSA-DTA, *n* = 47) mice.

Bakocevic et al. (15) showed that DT-induced basophil depletion at 4 d p.i. resulted in a modest reduction in *P. yoelii* 17X parasitemia. In our model of constitutive basophil depletion, overall *P. y. yoelii* 17XNL peripheral parasitemias were not significantly different in baso (−) mice compared with baso (+) mice at any time point (Fig. 1). These observations suggested that differences in phenotypes between baso (−) and baso (+) mice would not be influenced by or dependent on differences in asexual parasite burden.

### Basophil depletion increased intestinal permeability at days 4, 6, and 8 p.i. and altered ileal mastocytosis and MC activation relative to baso (+) controls

**FIGURE 2.**
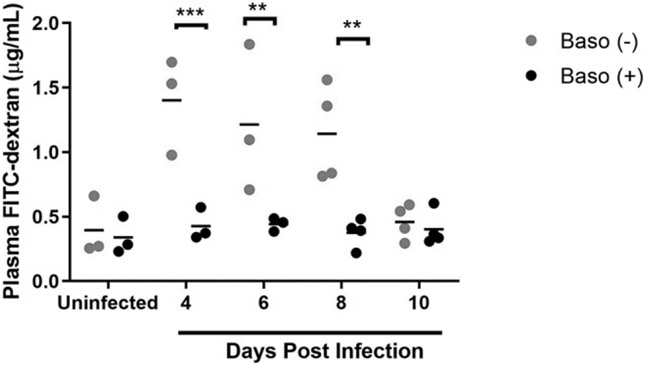
Baso (−) mice showed increased intestinal permeability relative to baso (+) mice at 4, 6, and 8 d postinfection. In vivo intestinal permeability was quantified by plasma FITC-dextran concentration following oral gavage of *P. y. yoelii* 17XNL–infected and uninfected mice of each genotype. Each dot represents a single mouse. Data were analyzed with a Kruskal–Wallis test followed by a Dunn’s multiple comparison test between genotypes at each time point. A *p* value < 0.05 was considered significant. **p < 0.01, ***p < 0.001.

**FIGURE 3.**
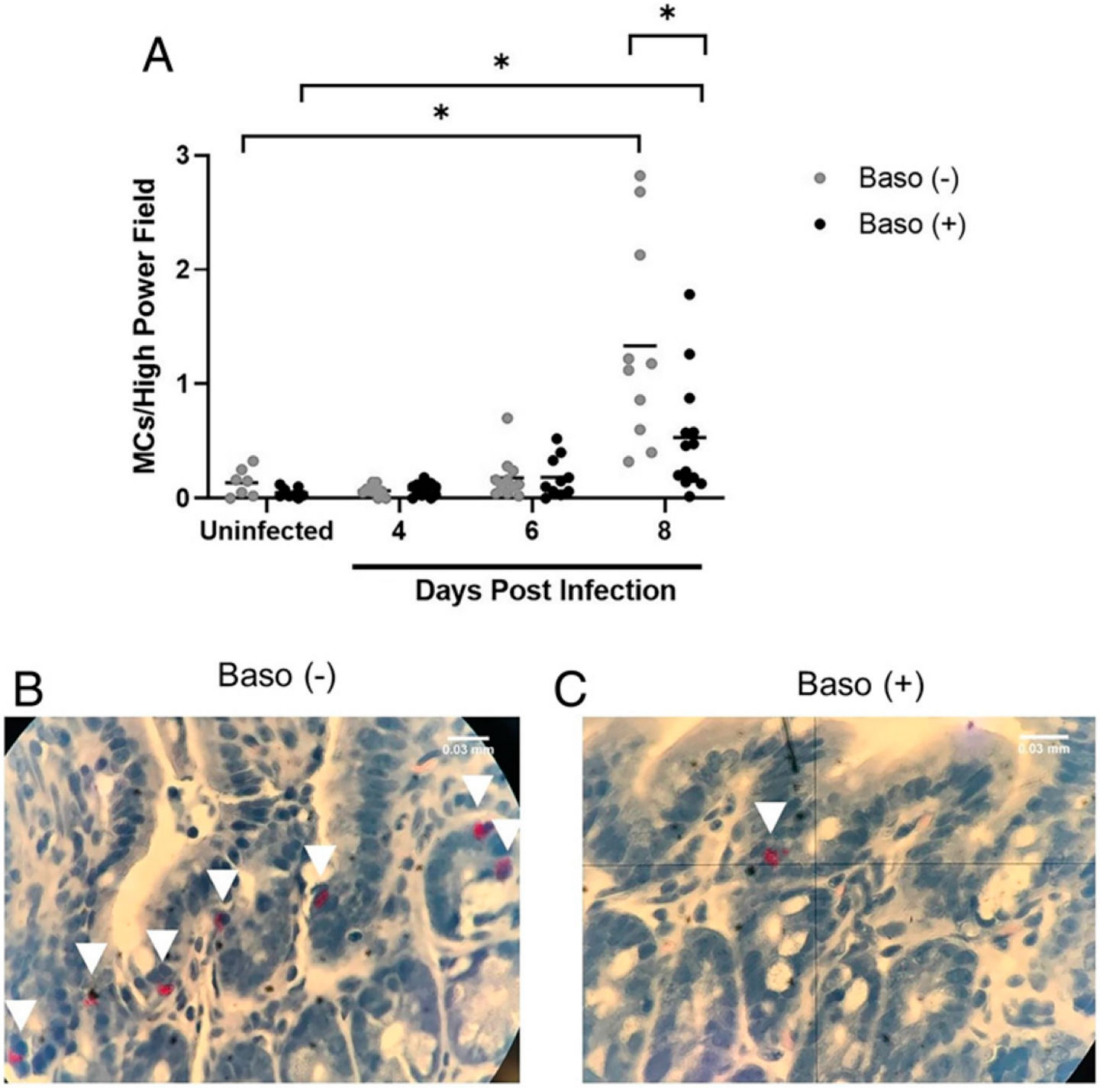
Baso (−) mice showed increased accumulation of ileal mast cells compared with baso (+) mice at 8 d postinfection. **(A)** Mean numbers of ileal mast cells (MCs) per high-powered field from NASDCE staining of sections from infected and uninfected mice of both genotypes. Each dot represents one mouse. These data were analyzed by a Brown–Forsythe ANOVA test followed by a Dunnett’s multiple comparisons test between genotypes at each time point. A *p* value < 0.05 was considered significant. *p≤ 0.05. **(B** and **C)** Stained MCs (pink cells indicated by white arrows) in the ileum of a baso (−) mouse (B) and a baso (+) mouse (C), both at 8 d postinfection.

Given that basophils are important sources of a number of factors that can modulate intestinal permeability, including IL-4 and histamine (22-24), we sought to assess the effect of basophil depletion on intestinal permeability following *P. y. yoelii* 17XNL infection. Baso (−) mice exhibited increased intestinal permeability to FITC-dextran at days 4, 6, and 8 p.i. relative to baso (+) mice (Fig. 2). By 10 d p.i., permeability in depleted and baso (+) mice returned to baseline. We previously showed that MCs accumulate in the intestine of malaria parasite–infected mice, with significantly increased numbers above uninfected control by days 4 and 8 p.i., and that MCs regulate permeability during infection (7-9). Based on these observations and increased intestinal permeability in baso (−) mice, we quantified ileal MCs following NASDCE staining of tissue from infected and uninfected mice. By 6 d p.i., both baso (−) and baso (+) mice trended toward an increase in ileal MCs relative to uninfected mice (Fig. 3A). By 8 d p.i., ileal MC numbers were significantly higher in baso (−) mice relative to baso (+) mice (Fig. 3).

**FIGURE 4.**
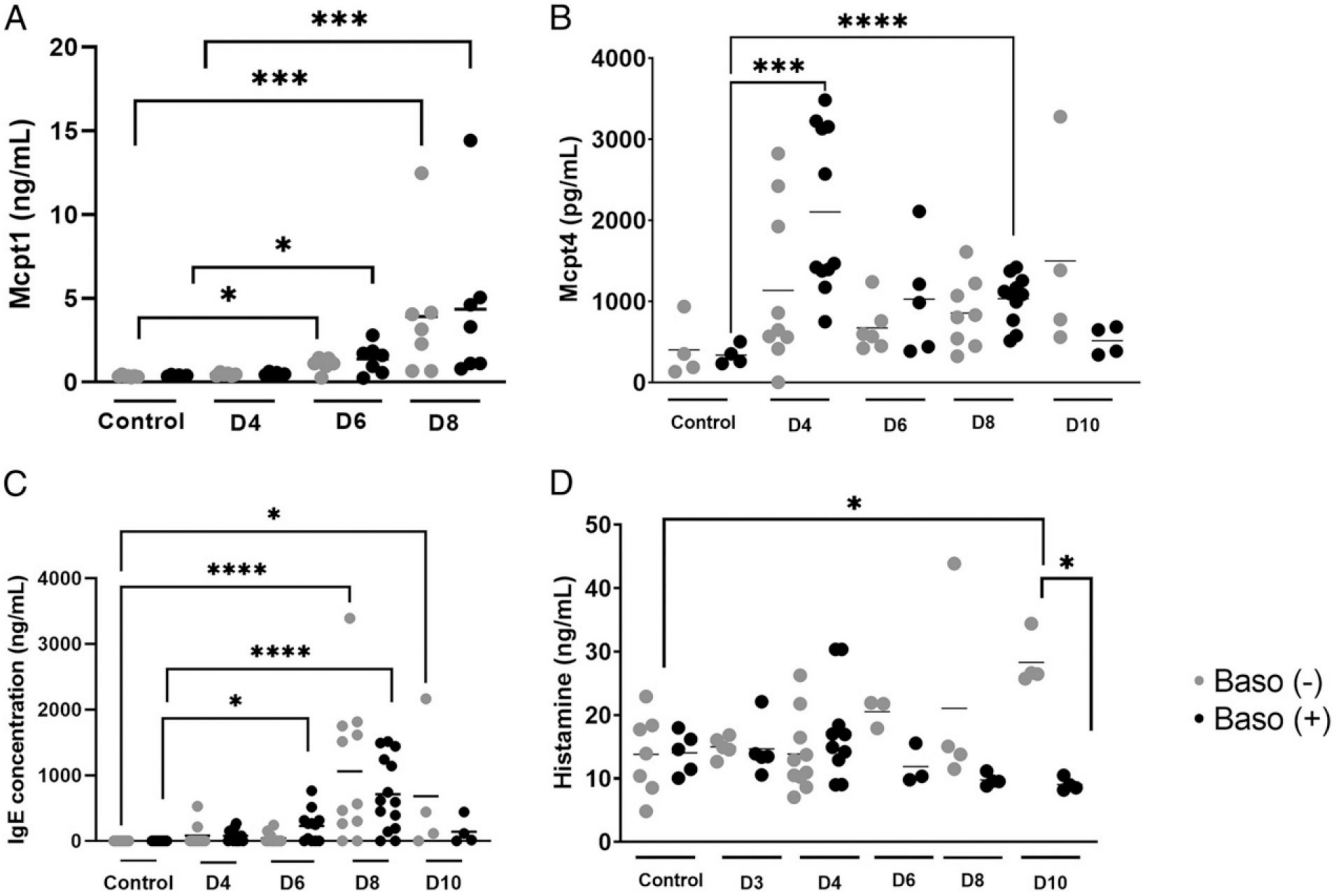
Plasma Mcpt1, Mcpt4, IgE, and histamine levels as determined by ELISA. **(A)** Plasma Mcpt1, **(B)** Mcpt4, **(C)** IgE, and **(D)** histamine as determined by ELISA in uninfected baso (−) mice and baso (+) mice, and at indicated days (D) postinfection in both genotypes. Data in (A) and (C) were analyzed with a Kruskal–Wallis test follows by a Dunn’s multiple comparison test between genotypes at each time point. Data in (B) and (D) were analyzed with a Brown–Forsythe and Welch ANOVA. A *p* value <0.05 was considered significant. *p < 0.05, ***p < 0.001, ****p < 0.0001.

In addition to MC recruitment in response to various stimuli, both resident and newly recruited MCs can be activated to release preformed and inducible mediators, which can act both locally and systemically to alter the host response to infection or inflammatory stimuli. In particular, MCs express a variety of cell surface receptors, including the high-affinity IgE receptor FcεRI, and receptors for cytokines, chemokines, pathogen-associated molecular patterns, as well as TLRs, which activate release of a variety of mediators following ligand interactions (25). Among these mediators are Mcpt1 and Mcpt4, which are inducibly produced by mucosal and connective tissue MCs, respectively (26, reviewed in Ref. 27), and are associated with increased intestinal permeability (28-31). Plasma IgE is an activator of basophils and MCs, while histamine is a potent effector synthesized by activated basophils and MCs. Relative to uninfected controls, levels of plasma Mcpt1 were increased by 6 d p.i., with higher levels by 8 d p.i. in both baso (−) and baso (+) mice (Fig. 4A). In contrast, plasma Mcpt4 levels were increased only in baso (+) mice relative to uninfected controls, with significant increases at 4 and 8 d p.i. that declined to uninfected control levels at 10 d p.i. (Fig. 4B). Expression patterns of MC proteases in baso (+) mice were similar to those previously reported in *P. y. yoelii* 17XNL-infected wild-type mice, which exhibited significantly increased plasma Mcpt1 on days 6 and 8 p.i. and significantly increased plasma Mcpt4 on days 4 and 8 p.i. (9). Relative to uninfected mice, IgE levels were significantly increased at day 6 p.i. in baso (+) mice and at day 8 p.i. in both baso (−) and baso (+) mice (Fig. 4C). Elevated IgE relative to control persisted in baso (−) mice through 10 d p.i. (Fig. 4C), suggesting that increased plasma IgE in baso (−) mice occurred later in infection relative to baso (+) mice. Circulating histamine levels were significantly increased in baso (−) mice relative to both uninfected controls and baso (+) mice at 10 d p.i. (Fig. 4D).

Taken together, these data confirmed previous observations (9) that MC activation during *P. y. yoelii* 17XNL infection is initiated by 4 d p.i. and extended our understanding of this activation by revealing that it is at least partially basophil-dependent (Fig. 4A, 4B). By 6 d p.i., ileal MCs trended higher than uninfected controls in both baso (−) and baso (+) mice, with significantly higher MC numbers in depleted mice at 8 d p.i. relative to both uninfected controls and baso (+) mice (Fig. 3A). MC activation might be sustained by later increases in IgE in depleted mice relative to uninfected controls as evidenced by significantly increased plasma histamine at 10 d p.i. in baso (−) mice relative to both baso (+) mice and uninfected controls. Collectively, these observations suggest that early activation of basophils in malaria dampens the host response to infection by decreasing and/or delaying various phenotypes, including MC activation-dependent intestinal permeability.

### Despite increased intestinal permeability and ileal MCs, basophil depletion did not alter blood 16S levels relative to baso (+) controls

**FIGURE 5.**
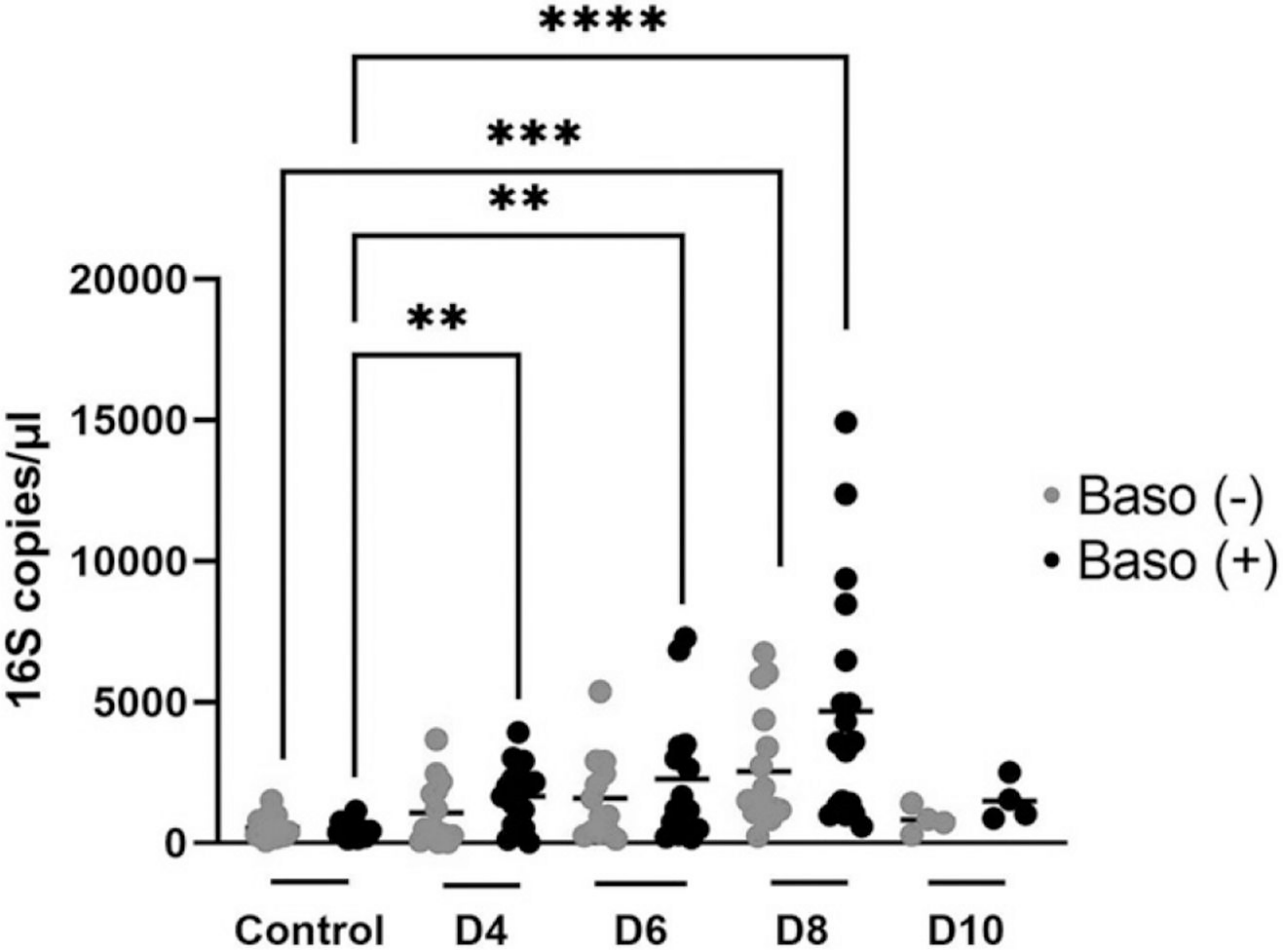
Basophil depletion did not alter circulating bacterial 16S levels at indicated days postinfection relative to baso (+) mice. Each dot represents a single mouse. Data were analyzed with a Kruskal–Wallis test followed by a Dunn’s multiple comparison test between genotypes at each time point. A *p* value <0.05 was considered significant. **p < 0.01, ***p < 0.001, ****p < 0.0001. D, day.

In our previous mouse model studies, we showed that MC activation is causally linked with the development of malaria-induced bacteremia (7, 8). Based on these observations, we sought to quantify bacterial 16S ribosomal DNA copies in blood, as a proxy for bacteremia, in baso (−) and baso (+) mice following parasite infection. As expected, 16S copy numbers rose with increasing parasitemia in both baso (−) and baso (+) mice. Despite significantly increased permeability to FITC-dextran on days 4, 6, and 8 p.i. in baso (−) mice relative to baso (+) mice (Fig. 2), 16S copy numbers were not significantly different between genotypes at any time point following infection (Fig. 5). However, baso (+) mice exhibited significantly higher 16S copy numbers 4 d earlier (4 d p.i.) than baso (−) mice (8 d p.i.) relative to uninfected controls, with 16S copy numbers declining back to uninfected control levels for both groups by 10 d p.i. (Fig. 5). Baso (−) mice, therefore, appeared to exhibit a delay in the development of malaria-induced bacteremia. Because bacteremia is the combined output of enteric bacterial translocation out of the gut and bacterial clearance, these findings suggested that the local and/or systemic immune response to translocating bacteria might be enhanced in baso (−) relative to baso (+) mice. To better understand the host immune response contributing to control of bacteremia, initiation of mastocytosis, and intestinal permeability, ileum and plasma samples were analyzed for cytokine and chemokines in both baso (−) and baso (+) mice at days 3, 4, 6, 8, and 10 p.i.

#### Ileal cytokine/chemokine levels.

**FIGURE 6.**
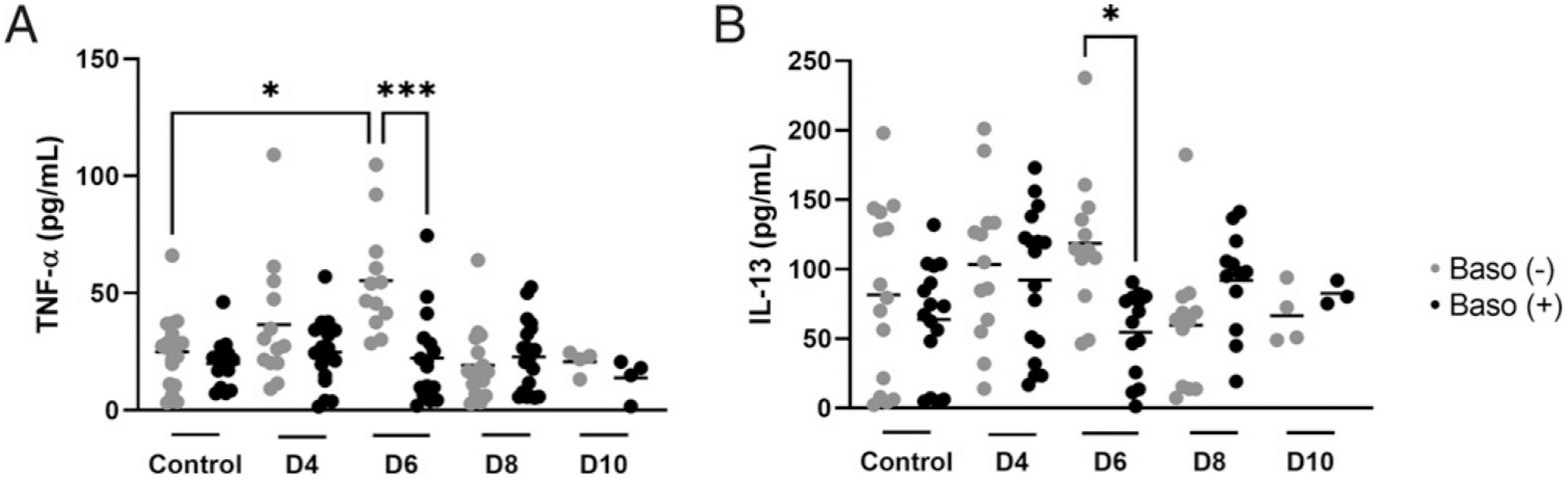
Ileal TNF-α and ileal IL-13 were increased at day 6 postinfection in baso (−) mice. (**A** and **B**) The *x*-axis represents days (D) postinfection and the *y*-axis represents ileal concentrations of TNF-α (A) and IL-13 (B). Each dot represents a single mouse. These data were analyzed with a Kruskal–Wallis test followed by a Dunn’s multiple comparison test between the baso (−) and baso (+) mice at each time point. A *p* value < 0.05 was considered significant. *p ≤ 0.05, ***p < 0.001.

**FIGURE 7.**
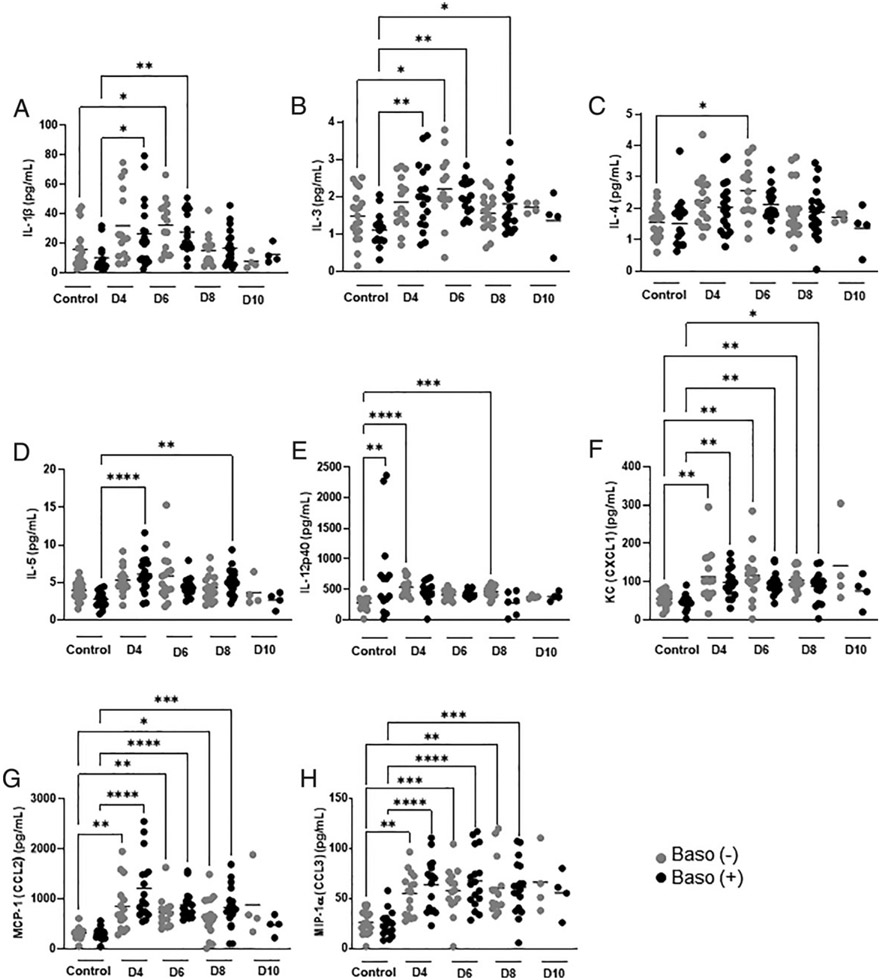
Ileal cytokines and chemokines in uninfected baso (−) mice and baso (+) mice and at indicated days postinfection in both genotypes. **(A–H)** The *y*-axis represents ileal concentrations of IL-1β (A), IL-3 (B), IL-4 (C), IL-5 (D), IL-12p40 (E), KC (F), MCP-1 (G), and MIP-1α (H). Each dot represents a single mouse. Normally distributed data (B) were analyzed with the Brown–Forsythe and Welch ANOVA. Nonnormal data (A and C–H) were analyzed with a Kruskal–Wallis test followed by a Dunn’s multiple comparison test between baso (−) and baso (+) mice at each time point. A *p* value <0.05 was considered significant. *p < 0.05, **p < 0.01, ***p < 0.001, ****p < 0.0001. D, day.

**FIGURE 8.**
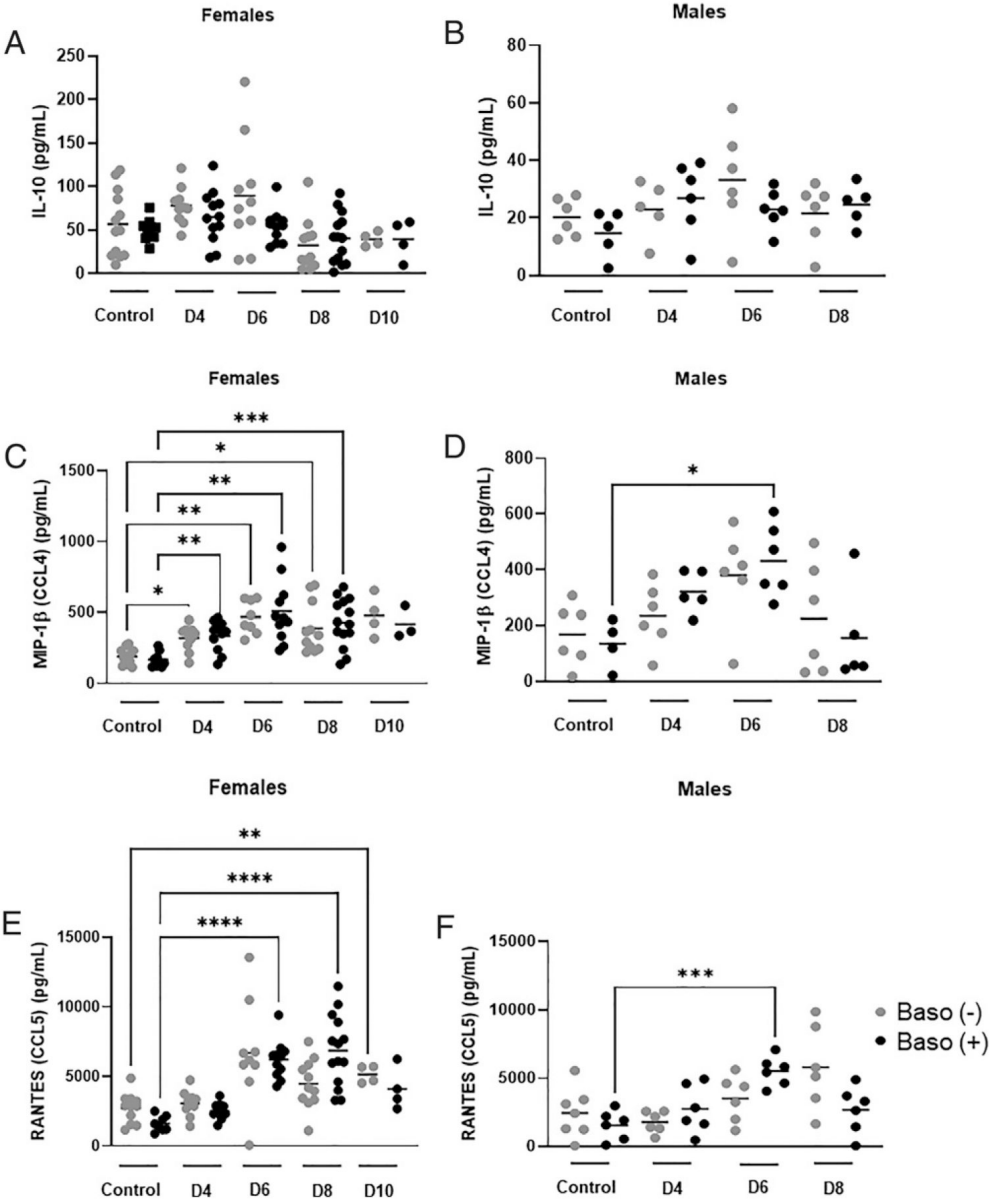
Sex-specific differences in ileum cytokines in uninfected baso (−) mice and baso (+) mice and at indicated days postinfection in both genotypes. **(A–F)** The *y*-axis represents ileal concentrations of IL-10 (A and B), MIP-1β (C and D) and RANTES (E and F). All data were normally distributed and analyzed with the Brown–Forsythe and Welch ANOVA. A *p* value <0.05 was considered significant. *p < 0.05, **p < 0.01, ***p < 0.001, ****p < 0.0001. D, day.

Two cytokines, TNF-α and IL-13, showed genotype-specific differences at 6 d p.i. in the ileum. Relative to baso (+) mice, baso (−) mice exhibited significantly increased ileal TNF-α, a proinflammatory cytokine, and IL-13, an immunoregulatory cytokine, at 6 d p.i., with TNF-α levels also significantly higher than for uninfected controls (Fig. 6A). The remaining ileal cytokines and chemokines showed distinct genotype-specific patterns over time, similar patterns over time for both genotypes, sex-specific differences, or no change relative to uninfected controls in either genotype (Figs. 7, 8, Supplemental Fig. 2).

Levels of several cytokines and chemokines differed relative to uninfected controls in temporal patterns that were genotype specific. For example, levels of ileal IL-1β, a proinflammatory cytokine, were increased above uninfected levels at 4 and 6 d p.i. in baso (+) mice, a pattern that was delayed and limited to 6 d p.i. in baso (−) mice (Fig. 7A). Similarly, the MC growth factor IL-3 was increased relative to uninfected levels from day 4 to 8 p.i. in baso (+) mice, but in baso (−) animals, IL-3 was significantly increased above uninfected levels only at day 6 p.i. (Fig. 7B). The Th2 polarizing cytokine IL-4 was increased above uninfected levels at day 6 p.i. in baso (−) mice only (Fig. 7C), which together with increased IL-13 and IL-3 at day 6 p.i. would represent a suite of factors associated with MC growth and activation consistent with increased ileal MCs in depleted mice at 8 d p.i. (Fig. 3A). The eosinophil stimulating factor IL-5 was increased relative to uninfected levels at days 4 and 8 p.i. only in baso (+) mice (Fig. 7D). Ileal IL-12p40, a subunit of IL-12 and IL-23 that, as a monomer, can attract macrophages (32), was significantly increased in uninfected baso (−) mice relative to baso (+) mice but was increased above uninfected levels at 4 and 8 d p.i. only in baso (−) mice (Fig. 7E).

The chemokines KC (a neutrophil chemoattractant), MCP-1 (a monocyte/macrophage chemoattractant), and MIP-1α (a chemoattractant for macrophages and T lymphocytes) were increased above uninfected control levels in both genotypes in the ileum at days 4, 6, and 8 p.i. (Fig. 7F-H). One ileal cytokine (IL-10, Fig. 8A, 8B) and two ileal chemokines (MIP-1β and RANTES, Fig. 8C-F) exhibited sex-specific differences. Mean levels of IL-10 were higher in the female mice of both genotypes compared with males (Fig. 8A, 8B), but levels of this cytokine were not increased above uninfected levels in either sex or genotype. Baso (+) male mice showed similar increases relative to uninfected controls in both MIP-1β and RANTES at day 6 p.i. (Fig. 8D, 8F), whereas females showed a different pattern. Female mice of both genotypes showed increased MIP-1β relative to uninfected mice at days 4, 6 and 8 p.i. (Fig. 8C). Female baso (+) mice showed increased RANTES relative to uninfected mice at days 6 and 8 p.i., whereas female baso (−) mice showed an increase only at day 10 p.i. (Fig. 8E). Levels of IL-1α, IL-2, IL-6, IL-9, IL-12p70, IL-17, eotaxin, G-CSF, GM-CSF, INF-γ, and IL-33 were not increased above uninfected levels in the ileum in either genotype (Supplemental Fig. 2).

#### Plasma cytokine/chemokine levels.

**FIGURE 9.**
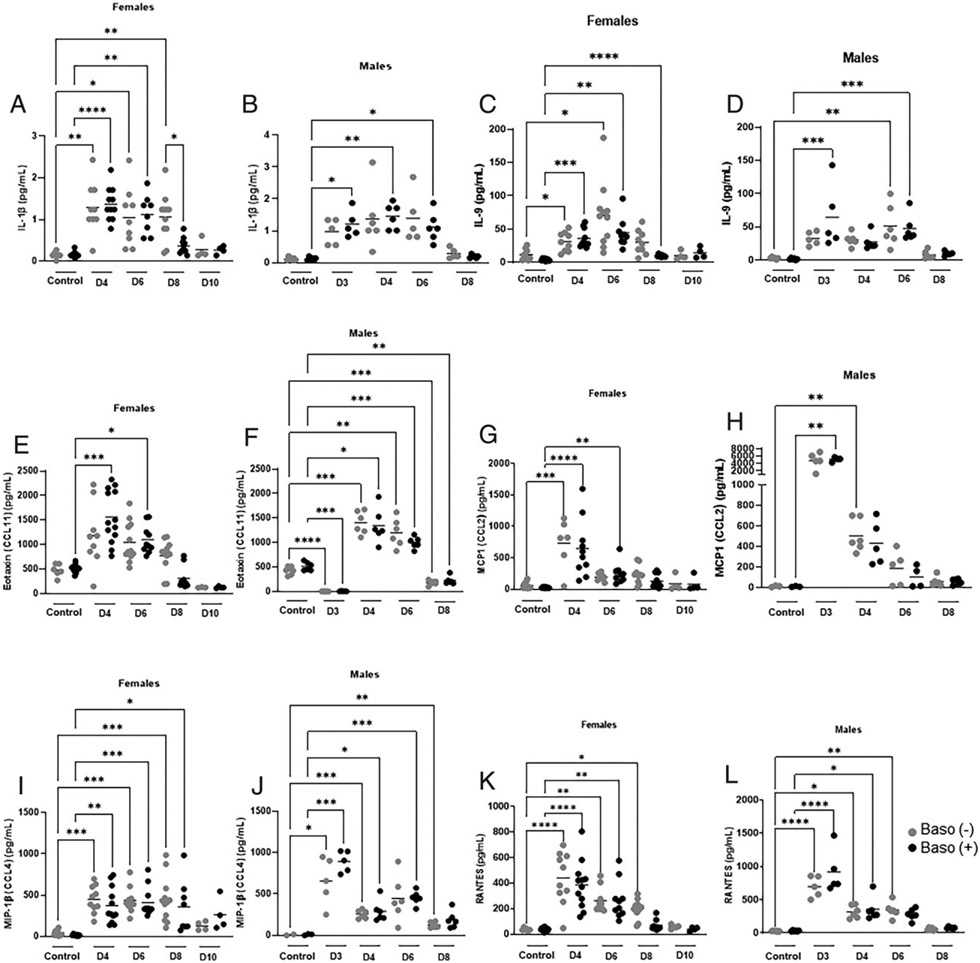
Sex-specific differences in plasma cytokines and chemokines in uninfected baso (−) mice and baso (+) mice and at indicated days postinfection in both genotypes. **(A–L)** The y-axis represents the plasma concentrations of IL-1β (A and B), IL-9 (C and D), eotaxin (E and F), MCP-1 (G and H), MIP-1β (I and J), and RANTES (K and L). Normally distributed data (B, C, F, H, and J) were analyzed with the Brown–Forsythe and Welch ANOVA. Nonnormal data (A, D, E, G, I, K, and L) were analyzed with the Kruskal–Wallis test followed by a Dunn’s multiple comparison test between genotypes at each time point. A *p* value < 0.05 was considered significant. *p < 0.05, **p < 0.01, ***p < 0.001, ****p < 0.0001. D, day.

**FIGURE 10.**
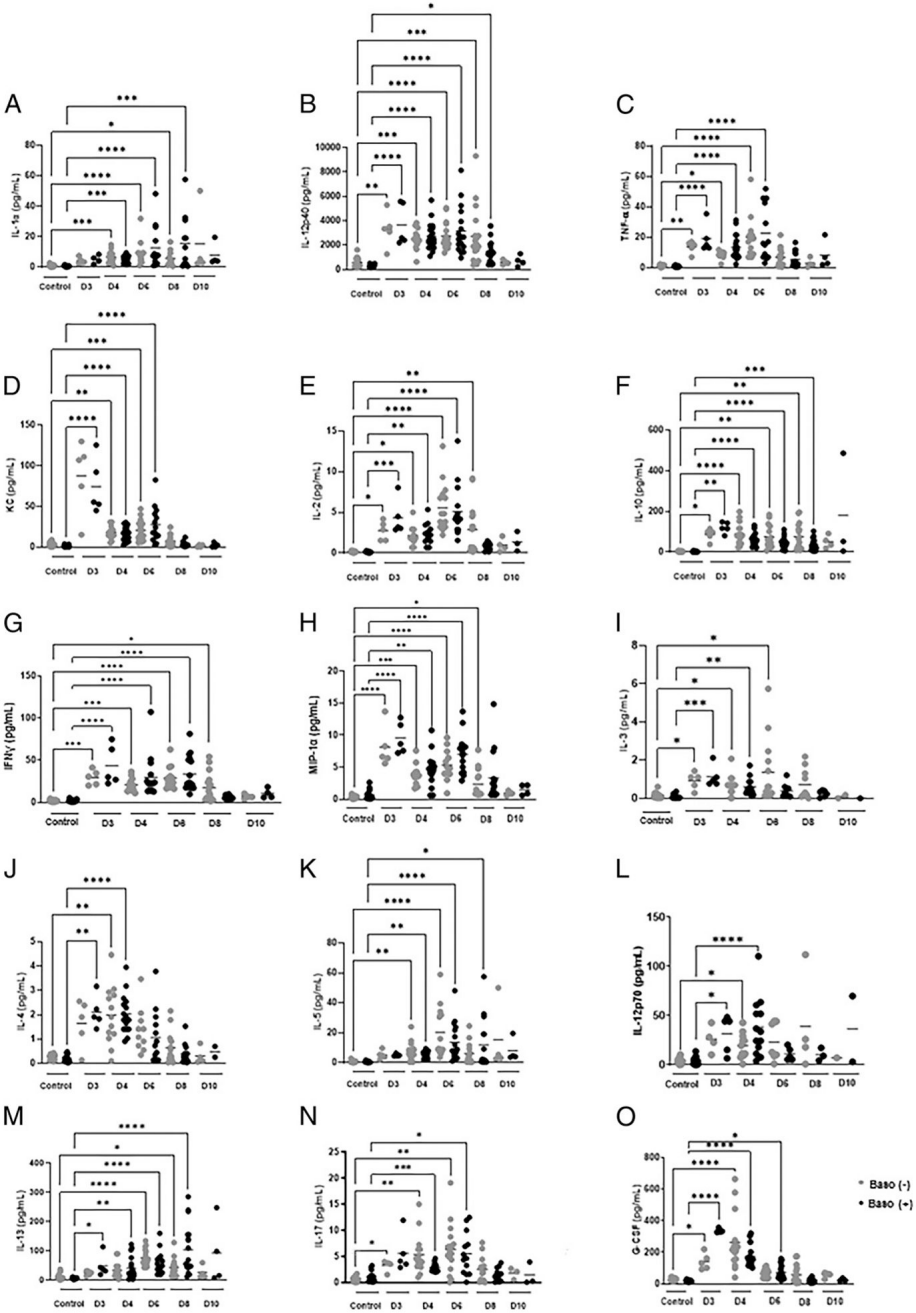
Plasma cytokines and chemokines without sex-specific differences in uninfected baso (−) mice and baso (+) mice at indicated days postinfection in both genotypes. **(A–O)** The y-axis represents the plasma concentrations of IL-1α (A), IL-12p40 (B), TNFa (C), KC (D), IL-2 (E), IL-10 (F), IFN-γ (G), M1Ρ−1α (H), IL-3 (I), IL-4 (J), IL-5 (K) IL-12p70 (L), IL-13 (M), IL-17 (N), and G-CSF (O). Normally distributed data (F and N) were analyzed with the Brown–Forsythe and Welch ANOVA. Nonnormal data (A–E, G–M, and O) were analyzed with a Kruskal–Wallis test followed by a Dunn’s multiple comparison test between the two genotypes at each time point. A *p* value <0.05 was considered significant. *p < 0.05, **p < 0.01, ***p < 0.001, ****p < 0.0001. D, day.

Plasma cytokines and chemokines showed sex- and/or genotype-specific increases relative to uninfected controls, distinct genotype-specific patterns over time, or similar patterns over time for both genotypes (Figs. 9, 10). Sex- and genotype-specific differences were noted for IL-1β, the MC survival factor IL-9, the eosinophil recruiting factor eotaxin, MCP1, MIP-1β, and the T cell-recruiting chemokine RANTES (Fig. 9). Both genotypes of female mice showed increased plasma IL-1β relative to uninfected levels on days 4 and 6 p.i., with this trend persisting only for depleted mice through day 8 p.i. (Fig. 9A). This difference was absent in males, where levels of plasma IL-1β were increased above uninfected levels only in baso (+) mice at 3, 4, and 6 d p.i. (Fig. 9B). Plasma IL-9 levels were increased relative to uninfected levels at days 4 and 6 p.i. in both baso (−) and baso (+) female mice, and this trend continued only in baso (+) females through 8 d p.i. (Fig. 9C). In contrast, baso (+) male mice exhibited increased plasma IL-9 relative to uninfected levels at days 3 and 6 p.i., although this pattern was evident only at 6 d p.i. in baso (−) male mice (Fig. 9D). Plasma eotaxin levels were significantly increased in baso (+) female mice with elevated levels relative to uninfected controls at days 4 and 6 p.i. (Fig. 9E). Male mice of both genotypes, in contrast, exhibited significantly reduced eotaxin relative to uninfected controls at days 3 and 8 p.i., and significant increases relative to uninfected controls on days 4 and 6 p.i. (Fig. 9F). In female mice, plasma MCP-1 was increased above uninfected levels at day 4 p.i. in baso (−) mice and at days 4 and 6 p.i. in baso (+) mice (Fig. 9G); in male mice, basophil depletion was associated with an increase at day 4 p.i. compared with an earlier increase at day 3 p.i. in baso (+) mice (Fig. 9H). Females of both genotypes showed similar increases relative to uninfected controls for plasma MIP-1β and RANTES at days 4, 6, and 8 p.i. (Fig. 9I, 9K), whereas male mice exhibited different patterns. MIP-1β levels were increased above uninfected levels at 3, 4, and 8 d p.i. in baso (−) males, whereas baso (+) male mice showed elevations at days 3, 4, and 6 p.i. (Fig. 9J). Baso (−) male mice exhibited elevations in RANTES relative to uninfected levels for longer (days 3, 4, and 6 p.i.) than did baso (+) male mice (days 3 and 4 p.i.) (Fig. 9L).

For those plasma cytokines and chemokines with no sex-specific differences over the course of infection, a majority, including IL-2, IL-10, IFN-γ, MIP-1α, IL-3, IL-4, IL-5, IL-12p70, IL-13, IL-17, and G-CSF, exhibited differing temporal patterns between genotypes. Four cytokines and chemokines, in contrast, showed the same patterns, including IL-1α, IL-12p40, TNF-α, and KC, that were increased in both genotypes relative to uninfected controls through 6–8 d p.i. with a return to baseline by 8–10 d p.i. (Fig. 10A-D). Among those with differing temporal patterns between genotypes, IL-2, IL-10, IFN-γ, and MIP-1α were significantly increased above uninfected levels in both genotypes at days 3, 4, and 6 p.i. (Fig. 10E-H), with increased levels persisting to day 8 p.i. in baso (−) mice (Fig. 10E-H). Plasma IL-3 was increased above uninfected levels at days 3, 4, and 6 p.i. in baso (−) mice and at days 3 and 4 p.i. in baso (+) mice (Fig. 10I). Plasma IL-4 was increased relative to uninfected levels at day 4 p.i. in baso (−) mice, while this increase was noted at day 3 p.i. in baso (+) mice (Fig. 10J). Plasma IL-5 was increased relative to uninfected levels at days 4 and 6 p.i. in baso (−) animals and at days 4, 6, and 8 p.i. in baso (+) mice (Fig. 10K). IL-12p70 was increased relative to uninfected levels only at day 4 p.i. in baso (−) mice, but at days 3 and 4 p.i. in baso (+) mice (Fig. 10L). Notably, IL-13 was increased relative to uninfected levels at all time points except day 10 p.i. in baso (+) mice, whereas it was increased only at days 4 and 8 p.i. in baso (−) mice (Fig. 10M). IL-17 was increased relative to uninfected levels at days 3–6 p.i. in baso (−) mice, but only at days 3 and 4 p.i. in baso (+) mice (Fig. 10N). The opposite pattern was observed for G-CSF, which promotes granulocyte proliferation and survival, where levels were increased relative to uninfected controls at days 3 and 4 p.i. in baso (−) mice and days 3–6 p.i. in baso (+) mice (Fig. 10O).

### Relative to baso (+) mice, baso (−) mice exhibited increased peripheral gametocytemia and were associated with increased intensity of *A. stephensi* infection following mosquito bloodfeeding

**FIGURE 11.**
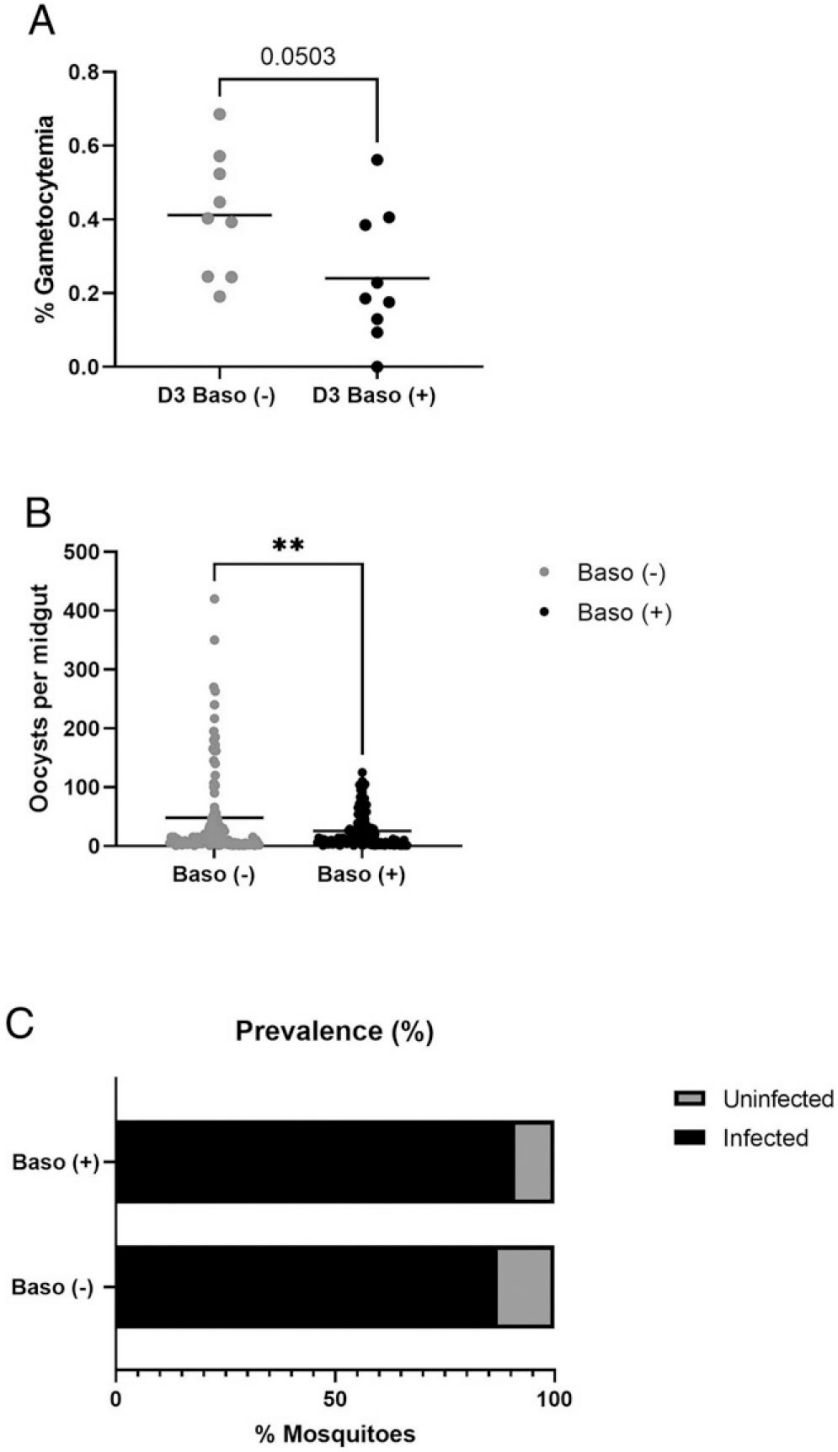
Basophil depletion altered *P. y. yoelii* 17XNL gametocytemia and parasite transmission to *A. stephensi*. **(A)** Peripheral gametocytemia at day 3 (D3) postinfection in baso (−) mice (*n* = 9) and baso (+) mice (*n* = 9). Each dot represents one mouse. Data were analyzed by using an unpaired t test with Welch’s correction. *p* = 0.0503. **(B)** *P. y. yoelii* 17XNL oocysts per midgut in *A. stephensi* fed on baso (−) mice and baso (+) mice at 3 d postinfection. Each dot represents one midgut with one or more oocysts. Data were analyzed using an unpaired *t* test with Welch’s correction. A *p* value <0.05 as considered significant. **p < 0.01. **(C)** The prevalence of mosquito infection following feeding on baso (−) and baso (+) mice. Mosquitoes were counted as infected when they had one or more midgut oocysts. Data were analyzed by aFisher’s exact test. A *p* value <0.05 was considered significant. *p* = 0.6839, not significant.

There is evidence to suggest that immune responses of the mammalian host can alter malaria parasite transmission to mosquitoes (33-36), observations that led us to examine the effects of basophils on this biology in our model. To this end, we examined gametocytemia and parasite transmission success to 3- to 5-d-old female *A. stephensi* using baso (−) and baso (+) infected mice. At 3 d p.i., the time point of peak infectivity of *P. y. yoelii* 17XNL to *A. stephensi,* there were no sex-specific differences in gametocytemia within genotypes (data not shown), so male mice were used for transmission studies. Notably, gametocytemia was increased in male baso (−) relative to male baso (+) mice (Fig. 11A), and this pattern was positively associated with increased parasite transmission success to *A. stephensi*. Specifically, mosquitoes that fed on infected male baso (−) mice developed significantly more oocysts per midgut compared with mosquitoes that fed on infected male baso (+) mice (Fig. 11B). Infection success was very high in both groups of mosquitoes, however, so the percentages of infected mosquitoes or infection prevalences were not significantly different between mosquitoes fed on infected baso (−) versus infected baso (+) mice (Fig. 11C).

Given our increasing understanding of the complexity and context dependence of basophil functions in not only bridging innate and adaptive immunity, but also in regulating neuroimmune interactions in the gut and general tissue health (37-40), we used network analysis to reveal basophil-associated networks between immune factors and key phenotypes (i.e., parasitemia, blood bacterial 16S copy numbers, gametocytemia, and capacity to infect mosquitoes) in infected baso (−) and baso (+) mice over time. To do this, we first constructed correlation matrices to identify significant positive and negative relationships between targets (parasitemia, gametocytemia, numbers of parasite oocysts in infected mosquitoes, and blood 16S copy numbers) and sources (ileal MC numbers, levels of plasma and ileal cytokines and chemokines, plasma IgE, plasma Mcpt1, Mcpt4, and histamine) for depleted and baso (+) mice over time following infection (Supplemental Fig. 3). Significant correlations (*p* ≤ 0.05) with fold changes >1.5 relative to uninfected controls were used to build interaction networks by day to evaluate changes over time for baso (−) and baso (+) mice.

**FIGURE 12.**
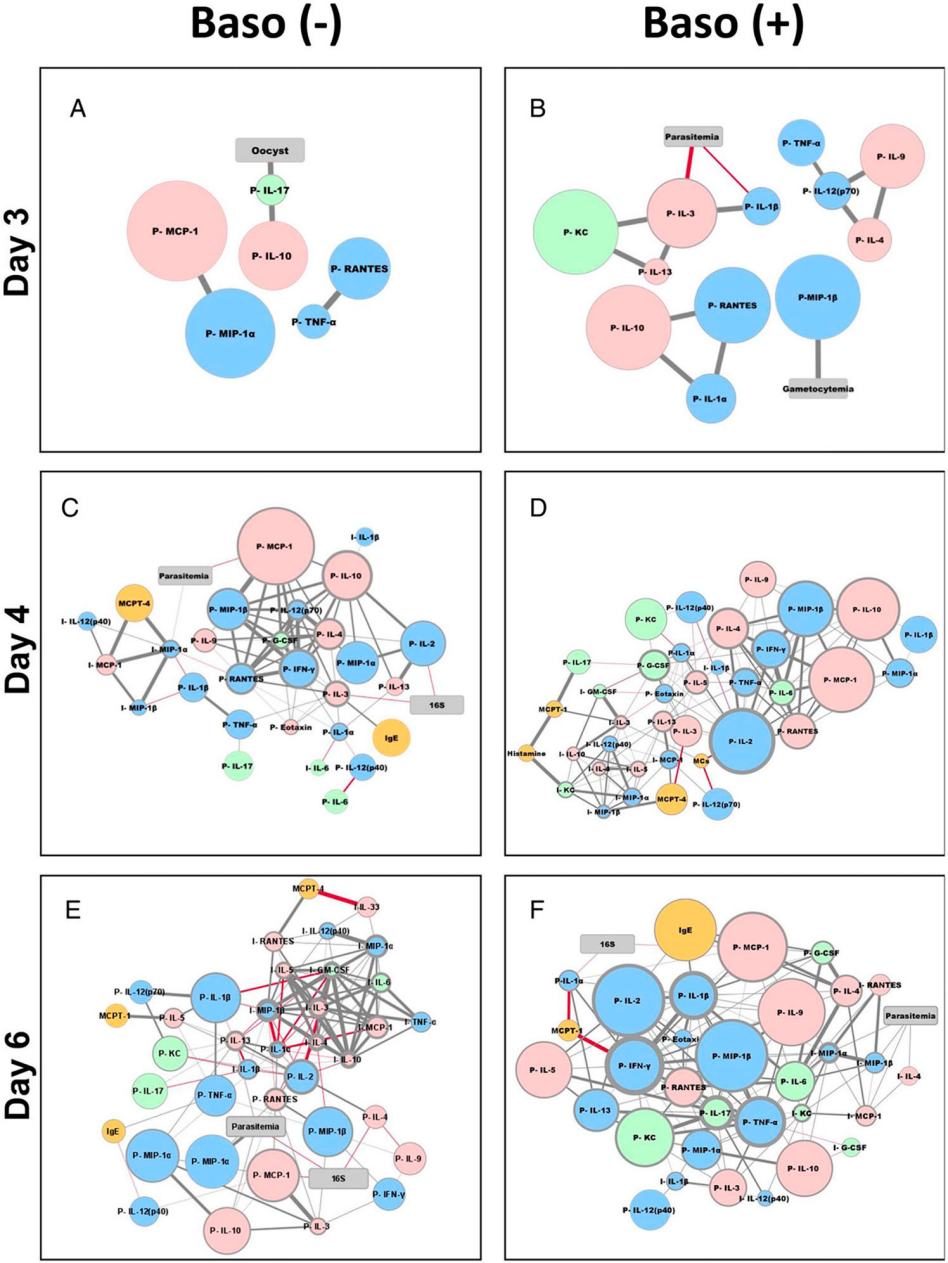
Network visualizations for baso (−) and baso (+) mice at days 3, 4, and 6 postinfection. **(A–F)** Network visualizations for baso (−) and baso (+) mice are shown at days 3 (A and B), 4 (C and D), and 6 (E and F) postinfection. Targets (gray rectangles: parasitemia, gametocytemia, numbers of mosquito oocysts, bacterial 16S copies in blood) and sources (circles: ileal MC numbers, plasma (P) and ileal (I) cytokines and chemokines, plasma IgE, plasma Mcpt1, Mcpt4, and histamine) were analyzed as described in the text for combined baso (−) male (*n* = 25) and female (*n* = 37) mice (left) and baso (+) male (*n* = 25) and female (*n* = 46) mice (right). Increasing circular node size reflects increasing fold change of a source calculated as level in infected baso (−) (*n* = 62) mice or baso (+) (*n* = 71) mice divided by level in uninfected baso (−) mice (*n* = 17) or baso (+) (*n* = 17), respectively. Blue nodes are type 1, pink nodes are type 2, and green nodes are type 17 cytokines and chemokines. MCs, IgE, Mcpt1, Mcpt4, and histamine are included as gold circles. Gray strokes correspond to positive correlations, and red strokes correspond to negative correlations. Increasing stroke width reflects increasing a Spearman’s correlation value. Increasing node outline thickness reflects an increasing number of connections with other nodes.

Day 3 p.i. coincided with studies of parasite transmission to *A. stephensi*. In baso (−) mice (Fig. 12A), parasite oocysts in *A. stephensi* were directly and strongly positively correlated with plasma IL-17, which was directly and strongly positively correlated with plasma IL-10. In baso (+) mice at day 3 p.i. (Fig. 12B), parasitemia was directly and negatively correlated with plasma IL-1β and IL-3, whereas MIP-1β was directly and strongly positively correlated with gametocytemia. The network in baso (−) mice was reduced in complexity relative to baso (+) mice, but both networks had similar proportions of type 1 (blue) and type 2 (red) cytokines and chemokines.

At day 4 p.i. in baso (−) mice (Fig. 12C), 16S copy numbers were directly and weakly negatively correlated with plasma IL-2 and IL-3, whereas parasitemia was weakly correlated with plasma MCP-1 (negatively) and ileal MIP-1α (positively). In baso (+) mice (Fig. 12D), 16S copy numbers and parasitemia were not significantly correlated with any factors in the network and, hence, absent from the network. Ileal MC numbers were negatively correlated with plasma IL-2 and plasma IL-12p70, and positively but weakly correlated with ileal IL-3 (Fig. 12D). Overall, type 2 and type 17 (green) cytokines and chemokines as well as MCs and MC-related factors (gold) were overrepresented in the baso (+) network at day 4 p.i. (Fig. 12D).

At day 6 p.i. in baso (−) mice (Fig. 12E), parasitemia and 16S copy numbers were directly, although weakly, negatively correlated. 16S copy numbers were also directly and weakly negatively correlated with plasma IL-4 and MCP-1, whereas parasitemia was directly and strongly positively correlated with plasma MIP-1α, and directly and weakly positively correlated with plasma IL-10. Mcpt4 was directly and strongly negatively correlated with ileal IL-33 levels, and positively and directly correlated with ileal RANTES levels. In baso (+) mice (Fig. 12F), parasitemia was directly and weakly positively correlated with ileal MIP-1α, MIP-1β, MCP-1, and IL-4. Bacterial 16S copy numbers in blood were directly and weakly negatively correlated with IgE and plasma IL-1α in baso (+) mice. Overall, there were more negative correlations, including multiple strong negative correlations, in the baso (−) network at 6 d p.i. (Fig. 12E).

**FIGURE 13.**
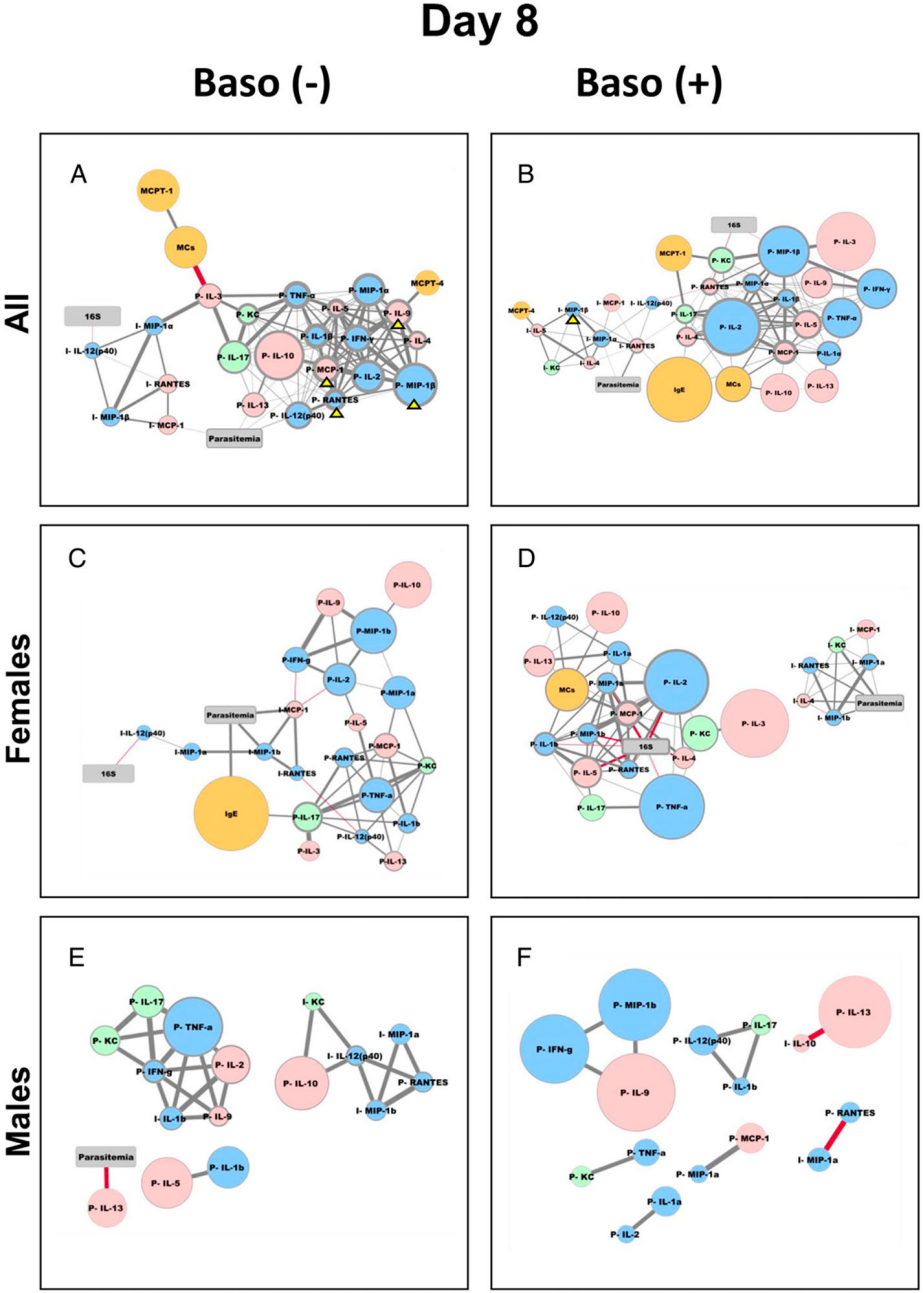
Network visualizations for baso (−) and baso (+) mice at day 8 postinfection with effects of mouse sex on the network. **(A–F)** Representations are as described in Fig. 9 for male and female baso (−) mice (left, A) and baso (+) mice (right, B), female mice only (C and D) and male mice only (E and F). Yellow arrowheads in the male and female mouse networks in (A) and (B) mark cytokines and chemokines that were specifically increased in female mice (Table I).

At day 8 p.i. in baso (−) mice (Fig. 13A), parasitemia was directly and positively correlated through three nodes to a large network of plasma factors and via one node to a smaller network of ileal factors. Mcpt1 was directly and positively correlated with ileal MCs, which, through a direct and strong negative correlation with plasma IL-3, bridged the plasma and ileal subnetworks. In contrast to the depleted network, the baso (+) network (Fig. 13B) revealed that parasitemia was connected via ileal RANTES and MIP-1α to a larger ileal network and that bacterial 16S was connected to a larger plasma network through plasma MIP-1β and KC. Hence, parasitemia and bacterial 16S copy numbers at day 8 p.i. were connected differentially to plasma and ileal subnetworks in depleted versus baso (+) networks.

In addition to these overall network relationships at day 8 p.i., we noted that immune factors that were increased to a greater degree in female mice (Table I) were driving strong positive correlations in baso (−) mice (Fig. 13A, yellow arrowheads) and, to a lesser extent, in baso (+) mice (Fig. 13B, yellow arrowhead). Accordingly, we reconstructed our day 8 p.i. networks using data from only female mice (Fig. 13C, 13D) or only male mice (Fig. 13E, 13F).

In female baso (−) mice at day 8 p.i. (Fig. 13C), the plasma and ileum subnetworks and the relationship with parasitemia were less distinct than in the combined male and female network (Fig. 13A). The numbers of strong central nodes (as inferred from node outline thickness) in the female network (Fig. 13C) were notably fewer than in the combined male and female analysis, perhaps as a result of fewer mice being included in the network, and the prominent Mcpt1/MC bridge between the ileal and plasma networks (Fig. 13A) was no longer evident in the female network. However, direct and positive correlations with parasitemia emerged for IgE and ileal MCP-1 in the female baso (−) network (Fig. 13C). The direct and negative correlation between bacterial 16S copy numbers and ileal IL-12p40 observed in the combined male and female network (Fig. 13A) remained unchanged in the female baso (−) mouse network (Fig. 13C), suggesting that this correlation is female-driven.

In female baso (+) mice at day 8 p.i. (Fig. 13D), the ileum and plasma subnetworks were entirely separate; however, as in the combined male and female analysis (Fig. 13B), parasitemia was correlated with the ileum subnetwork and 16S copy numbers were correlated with the plasma subnetwork. Parasitemia remained directly and positively correlated with ileal MIP-1α and RANTES but was also newly and directly positively correlated with ileal KC, IL-4, and MIP-1β in the female network (Fig. 13D). 16S copy numbers were correlated with a larger number of host factors in the female, baso (+) network (Fig. 13D) relative to the combined male and female network (Fig. 13B), including negative correlations with plasma TNF-α, IL-2, MCP-1, IL-5, IL-1β, IL-4, and RANTES. In contrast to the highly complex networks in the female mice at day 8 p.i., male mice of both genotypes were represented by greatly reduced networks (Fig. 13E, 13F). In baso (−) males (Fig. 13E), correlations formed subnetworks that were less distinctly ileum or plasma biased than in females. Bacterial 16S copy numbers were absent from the male, baso (−) network, and only IL-13 was directly and strongly negatively correlated with parasitemia in this network (Fig. 13E). Interestingly, the male, baso (+) mouse network (Fig. 13F) did not include correlations with parasitemia or 16S copy numbers, but rather was comprised only of pairs or groups of three cytokines, with a bias toward plasma-associated factors.

## DISCUSSION

Basophils have often been thought of as circulating MCs due to their phenotypic similarities and involvement with allergic inflammation. However, functional analyses with blocking Abs (41, 42) and mutant mouse lines (17, 43, 44) have enabled improved characterization of unique functions of basophils in homeostasis and disease (42, 45, 46). Although they function as effector cells during allergic responses, basophils can also be protective against tissue damage and pathology following helminth infection (47). In this context, the absence of basophils resulted in an overactive inflammatory response and increased IL-5 and IL-13 production by group 2 innate lymphoid cells in the lungs of mice infected with *Nippostrongylus brasiliensis* (47). In a somewhat similar vein, our data suggest that, in the context of malaria-induced mastocytosis and increased intestinal permeability, basophils may actually be protective against allergic inflammation in the gut, rather than a contributor to it. Baso (−) mice exhibited increased intestinal permeability at days 4, 6, and 8 p.i. (Fig. 2) and greater MC accumulation in the ileum at 8 d p.i. (Fig. 3A) relative to baso (+) mice. Although the higher permeability at day 8 p.i. can be at least partially explained by increased MC numbers at this time point, increased intestinal permeability also preceded MC influx. Céspedes et al. (19) found that Mcpt4 was also associated with protection against malaria-induced intestinal permeability, and whereas baso (+) mice in this study showed a significant increase in plasma Mcpt4 relative to uninfected levels, baso (−) mice did not (Fig. 4B).

Despite increased intestinal permeability and MC numbers in baso (−) relative to baso (+) mice during the course of infection, bacterial 16S copy numbers in blood were not significantly different between genotypes at any time point, with a trend toward lower copy numbers in depleted mice (Fig. 5). We propose that this trend could be at least partially explained by differences in ileal and plasma cytokine levels. For example, ileal TNF-α and IL-13 were significantly increased at 6 d p.i. in baso (−) mice (Fig. 6). Matsukawa et al. (48) reported that IL-13 did not alter bacterial loads in a sepsis model, but its positive association with increased epithelial permeability has been well documented (49, 50, reviewed in Ref. 51). IL-13 can be produced by several cell types in the intestine, including lymphocytes, NK cells, NKT cells, basophils, and MCs (52). TNF-α, in contrast, can increase paracellular permeability by disrupting ZO-1 and occludin-1 (53, 54) and is an important contributor to host defense against bacterial infections (55, 56, reviewed in Ref. 57). In the intestine, cells of the monocyte lineage including macrophages (58) are the major source of TNF-α (59), but intestinal MCs are also an important source of TNF-α following IgE receptor crosslinking (60). In a mouse model of cecal ligation and puncture, basophil-derived TNF-α contributed to elevated i.p. and serum levels of this cytokine, enhanced bacterial clearance, and reduced morbidity and mortality (39), patterns not observed in our nonlethal model. However, further studies involving the blockade of IL-13 and TNF-α will be necessary to validate the roles of these cytokines in the phenotypes observed in our studies. Additionally, IL-12p40 (Fig. 7E) was elevated above uninfected levels in baso (−) mice at days 4 and 8 p.i. in the ileum, whereas baso (+) mice did not show this increase. Given that IL-12p40 is a chemoattractant for macrophages (32) and can be produced by APCs and dendritic cells in response to bacterial stimuli (61-64), these data suggest the possibility that the immunological intestinal barrier of baso (−) animals may be more effective in controlling bacterial translocation. Network analysis of baso (−) mice at day 8 p.i. (Fig. 13A, 13B) showed a negative correlation between IL-12p40 and bacterial 16S copy numbers in the ileal subnetwork, whereas parasitemia was positively correlated with cytokines and chemokines predominantly in the plasma subnetwork in baso (−) mice. Importantly, note that network analysis highlights potential interactions to inform hypotheses to be tested in future studies. For example, differences in the day 8 networks between baso (−) and baso (+) mice suggest that baso (+) mice are unable to control bacterial translocation at the level of the ileum and, in turn, control of bacteremia has transitioned to a systemic response. It is also noteworthy that femaleness has a strong influence on the host response networks (Fig. 13C-F). This could be due, at least in part, to the fact that more female mice were used in these studies. However, there is evidence suggesting a female bias in MC-associated diseases such as systemic anaphylaxis (65) and irritable bowel syndrome (66). Furthermore, Mackey et al. (67) found sexual dimorphism in mouse bone marrow–derived MCs, noting that female bone marrow–derived MCs had “increased synthesis, storage, and release of MC granule-associated mediators that is independent of the estrous cycle.”

Perhaps the most interesting and unexpected outcomes of these studies were the impacts of basophils on gametocytemia and parasite transmission to mosquitoes. Barry et al. (36) noted that chronic cases of malaria likely contribute more to mosquito infection than do incident malaria cases, and that infection is less likely to be successful when gametocyte donors were symptomatic. Although the identity of these factors is unknown in symptomatic donors, Barry et al. (36) commented that this finding is consistent with animal models in which inflammatory cytokines and their intermediates have been shown to have gametocidal properties. TNF-α and IFN-γ, for instance, have both previously been demonstrated to be involved with gametocyte killing in the mammalian host (68, 69), with TNF-α being shown to impact transmission in rodent models (70). Macrophages in culture have been shown to phagocytose gametocytes and produce TNF-α, MIP-2, and NO in response to late-stage gametocytes, suggesting innate immune involvement in controlling sexual stage parasites (71). In our network analyses, plasma IL-17 and IL-10 in baso (−) mice at 3 d p.i. were strongly positively correlated with the presence of oocysts in *A. stephensi* (Fig. 12A). In contrast, oocysts were absent from the baso (+) network, parasitemia was negatively correlated with plasma IL-3 and IL-1β, and gametocytemia was strongly positively correlated with plasma MIP-1β at 3 d p.i. (Fig. 12B). Notably, IL-17 and IL-10 can significantly enhance myelopoiesis and erythropoiesis in mouse spleen and bone marrow (72-74). In this context, the network correlations of oocysts with IL-17 and IL-10 may, therefore, be related to enhanced gametocytogenesis and observed increased circulating gametocytes in baso (−) mice (Fig. 11A). Specifically, splenic early reticulocytes have been shown to provide a cryptic niche for sexual development of *P. berghei* ANKA (75), biology that could be relevant to reticulocyteprone *P. y. yoelii* 17XNL as well (76). In considering elements of the baso (+) network at 3 d p.i., elevated IL-3 has been reported to suppress protective immunity to *P. berghei* NK65 (77), with elevated MIP-1β in the context of elevated RANTES associated with protection against severe falciparum malaria (78) and elevated levels of IL-1β associated with lower childhood parasitemia and protection against severe falciparum malarial anemia (79, 80). While human and mouse responses in malaria are substantially distinct, increases in these host factors could help to explain the type 2– and type 17–skewed responses in baso (+) mice at 4 d p.i. (Fig. 12D) that perhaps function early in infection to preserve both parasite survival and host health. We are currently focused on examining a variety of specific host factors in our model system to identify potential mechanisms of basophil-mediated control of gametocytemia and parasite transmission.

Taken together, to our knowledge, these findings demonstrate novel and multifaceted roles of basophils in the context of malaria. We are aware that these studies have several limitations. First, baso (−) mice are deficient in basophils from birth. Although we did not observe any significant differences in immune factors between the two genotypes in the absence of infection, it is possible that this lifelong deficiency could have an impact on the immune response. Furthermore, we have not defined mechanisms for the phenotypes observed in these studies. However, these studies provide critical insights for testing a number of novel hypotheses supported by our observations. Future directions will focus on defining basophil-dependent mechanisms for protective phenotypes related to intestinal permeability and MC activation in malaria, as well as defining how basophils impact patterns of gametocytemia and parasite transmission to mosquitoes.

## Supplementary Material

Supplemental figures

## Figures and Tables

**TABLE I.** Sex-specific cytokine differences

Cytokine	Plasma/Ileum	Days p.i.	Genotype	Direction
IL-1β	Plasma	8	Baso (−)	Females higher
IL-9	Plasma	8	Baso (−)	Females higher
MCP-1	Plasma	8	Baso (−)	Females higher
RANTES	Plasma	8	Baso (−)	Females higher
Eotaxin	Plasma	8	Baso (−)	Females higher
MIP-1β	Plasma	4, 8	Baso (−)	Females higher
MIP-1β	Ileum	8	Baso (+)	Females higher
RANTES	Ileum	8	Baso (+)	Females higher
IL-10	Ileum	Control	Baso (+)	Females higher
IL-10	Ileum	4	Baso (−)	Females higher

Summary of sex-specific differences in cytokines and chemokines across genotypes, days postinfection (p.i.), and tissue (plasma and ileum).

## Abbreviations used in this article:

DT: diphtheria toxin
HPF: high-power field
MC: mast cell
Mcpt: mast cell protease
NASDCE: naphthol AS-D chloroacetate esterase
