# Supplementary material for: Basophil Depletion Alters Host Immunity, Intestinal Permeability, and Mammalian Host-to-Mosquito Transmission in Malaria
Source: Immunohorizons. Author manuscript; Available in PMC 2023 Mar 1. (PMC9977168; doi:10.4049/immunohorizons.2200055)
Supplement: Supplemental figures [file NIHMS1870867-supplement-Supplemental_figures.docx]

**Supplementary Figures**

**B**
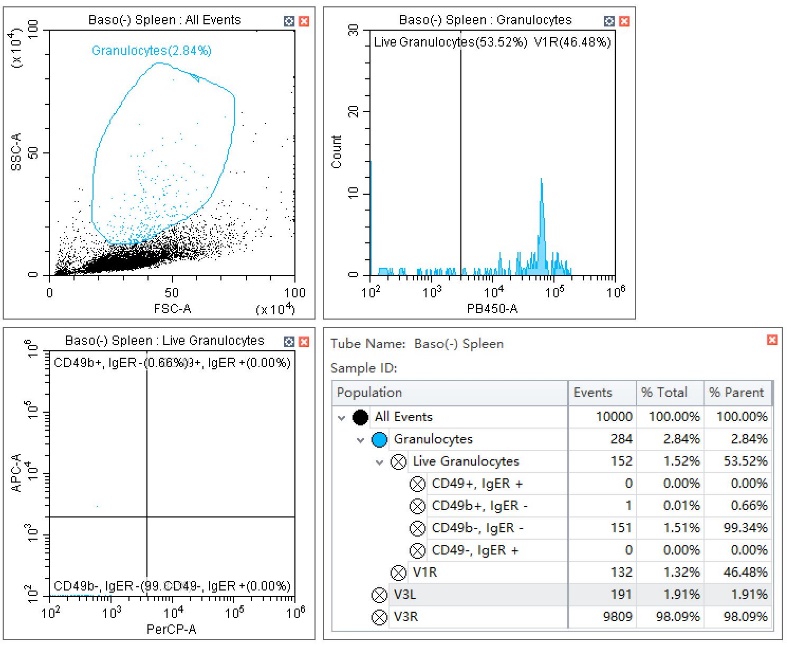


**A**
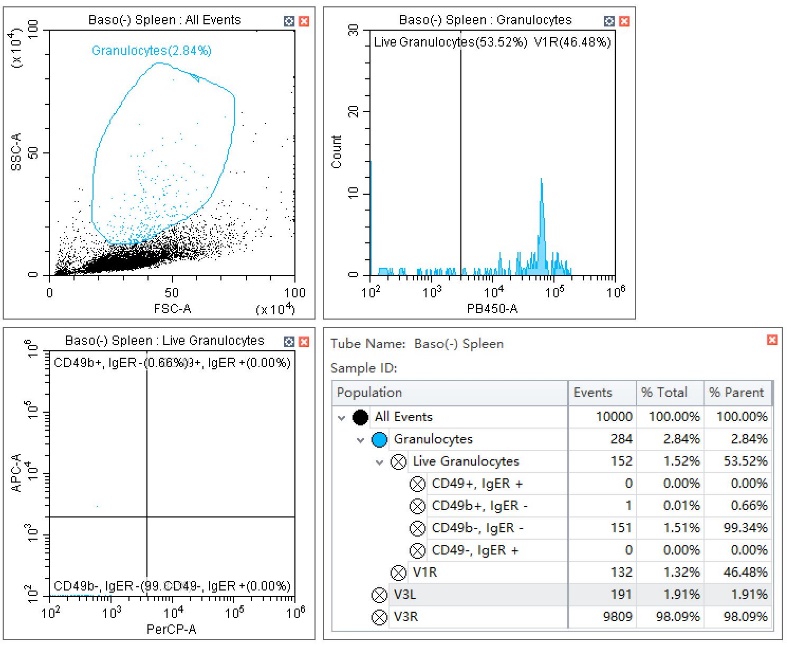


**
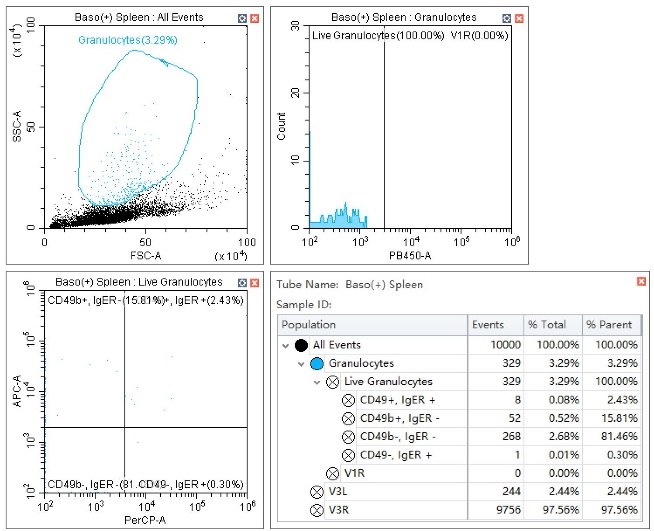

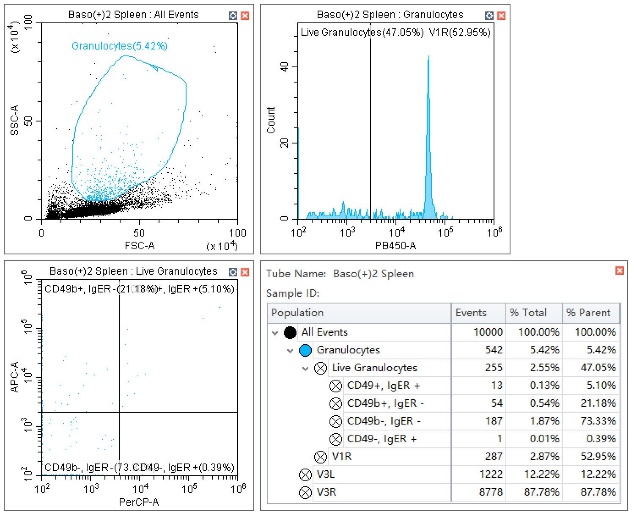
**

**C**
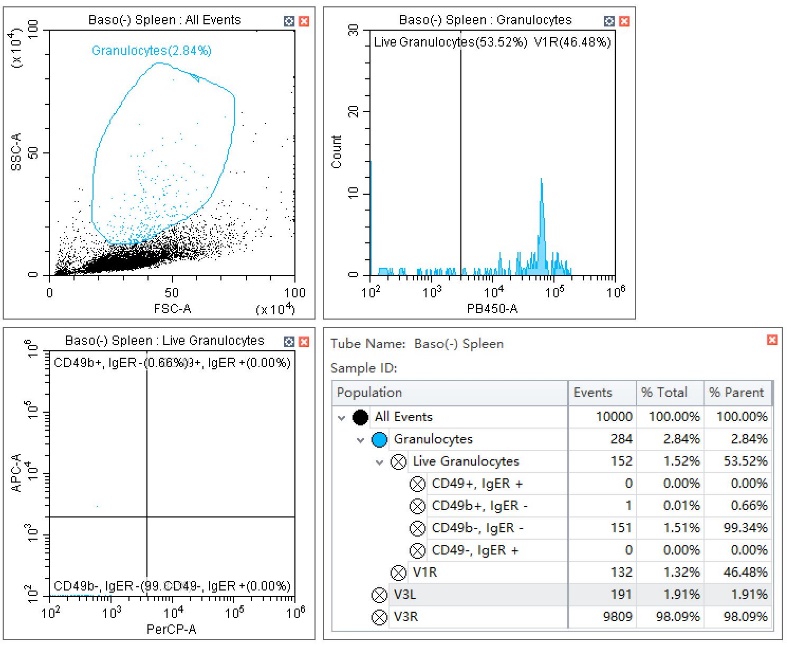


**
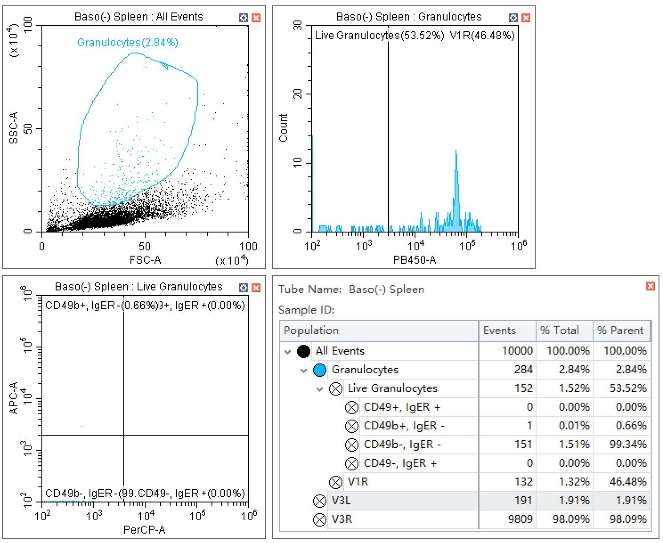

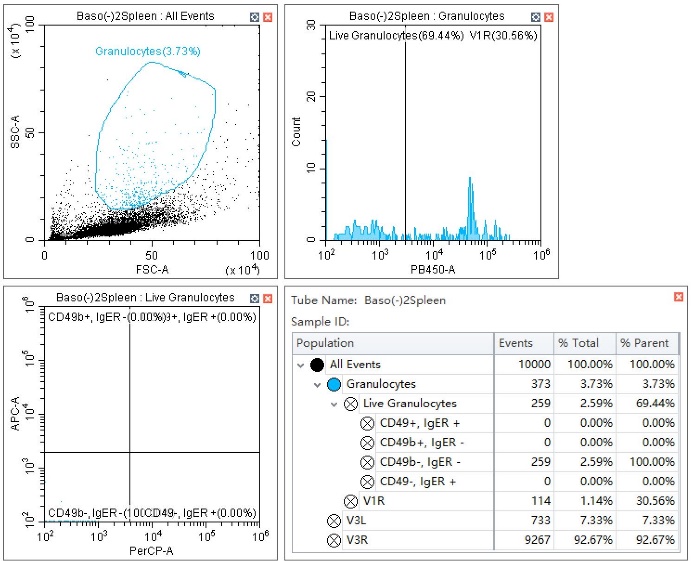
**

**D**
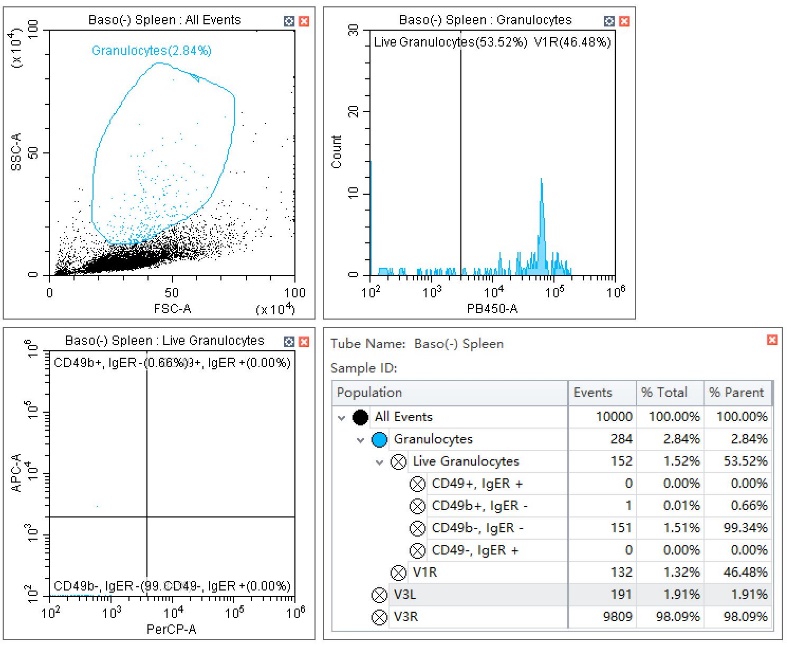


**Figure S1.** Flow cytometry gating strategy to confirm depletion of basophils in Basoph8 x ROSA-DTα mice. After gating out dead cells (DAPI/PB450+), FCεRI (APC) and CD49b (PerCP) were used to define the basophil population in the spleen. (A&B) In non-depleted mice, basophils (defined as FCεRI, CD49b double positive cells) account for 8 events (2.43% of the live granulocyte population) in (A) and 13 events (5.1% of the live granulocyte population) in (B). (C & D) In basophil-depleted animals, FCεRI, CD49b double positive cells were not detected.

**Figure S2.** Ileal cytokines and chemokines in control uninfected basophil-depleted Baso (-) mice and non-depleted Baso (+) mice and at indicated days (D) post-infection in both genotypes. The y-axis represents ileal concentrations of IL-1β (A), IL-3 (B), IL-4 (C), IL-5 (D), IL-12p40 (E), KC (F), MCP-1 (G), and MIP-1α (H). Each dot represents a single mouse. Normally distributed data (B) were analyzed with the Brown-Forsythe & Welch ANOVA. Non-normal data (A, C-H) were analyzed with Kruskal-Wallis test followed by Dunn’s multiple comparison between the basophil-depleted and non-depleted mice at each time point. P values of < 0.05 were considered significant. *, P ≤ 0.05, **, P ≤ 0.01, ***, P < 0.001, ****, P ≤ 0.0001.

**Figure S3.** Sex-specific differences in ileum cytokines in control uninfected basophil-depleted Baso (-) mice and non-depleted Baso (+) mice and at indicated days (D) post-infection in both genotypes. (A to F) The y-axis represents ileal concentrations of IL-10 (A and B), MIP-1β (C, D) and RANTES (E, F). All data were normally distributed and analyzed with the Brown-Forsythe & Welch ANOVA. P values of < 0.05 were considered significant. *, P ≤ 0.05. **, P <0.01, ***, P < 0.001, ****, P < 0.0001.

**Figure S4**. Ileal cytokines and chemokines that were unchanged relative to levels in control uninfected basophil-depleted Baso (-) mice and non-depleted Baso (+) mice and at indicated days (D) post-infection in both genotypes. The y-axis represents the ileal concentrations of IL-1α (A), IL-2 (B), IL-6 (C), IL-9 (D), IL-12p70 (E), IL-17 (F), eotaxin (G), G-CSF (H), GM-CSF (I), IFNγ (J) and IL-33 (K). Normally distributed data (B, D, E and H-J) were analyzed with the Brown-Forsythe & Welch ANOVA. Non-normal data (A, C, F, G and K) were analyzed with the Kruskal-Wallis test followed by Dunn’s multiple comparisons. P values of < 0.05 were considered significant.

**Figure S5.** Sex-specific differences in plasma cytokines and chemokines in control uninfected basophil-depleted Baso (-) mice and non-depleted Baso (+) mice and at indicated days (D) post-infection in both genotypes. (A to L) The y-axis represents the plasma concentrations of IL-1β (A and B), IL-9 (C and D), eotaxin (E and F), MCP-1 (G and H), MIP-1β (I and J) and RANTES (K and L). Normally distributed data (B, C, F, H, and J) were analyzed with the Brown-Forsythe & Welch ANOVA. Non-normal data (A, D, E, G, I, K, L) were analyzed with the Kruskal-Wallis test followed by Dunn’s multiple comparison between genotypes at each time point. P values of < 0.05 were considered significant. *, P ≤ 0.05, **, P <0.01, ***, P < 0.001, ****, P < 0.0001.

**Figure S6.** Plasma cytokines and chemokines without sex-specific differences in control uninfected basophil-depleted Baso (-) mice and non-depleted Baso (+) mice at indicated days (D) post-infection in both genotypes. (A to O) The y-axis represents the plasma concentrations of IL-1α (A), IL-12p40 (B), TNFα (C), KC (D), IL-2 (E), IL-10 (F), IFNγ (G), MIP-1α (H), IL-3 (I), IL-4 (J), IL-5 (K) IL-12p70 (L), IL-13 (M), IL-17 (N) and G-CSF (O). Normally distributed data (F and N) were analyzed with the Brown-Forsythe & Welch ANOVA. Non-normal data (A-E, G-M, O) were analyzed with the Kruskal-Wallis test followed by Dunn’s multiple comparison between the two genotypes at each time point. P values of < 0.05 were considered significant. *, P ≤ 0.05, **, P <0.01, ***, P < 0.001, ****, P < 0.0001.


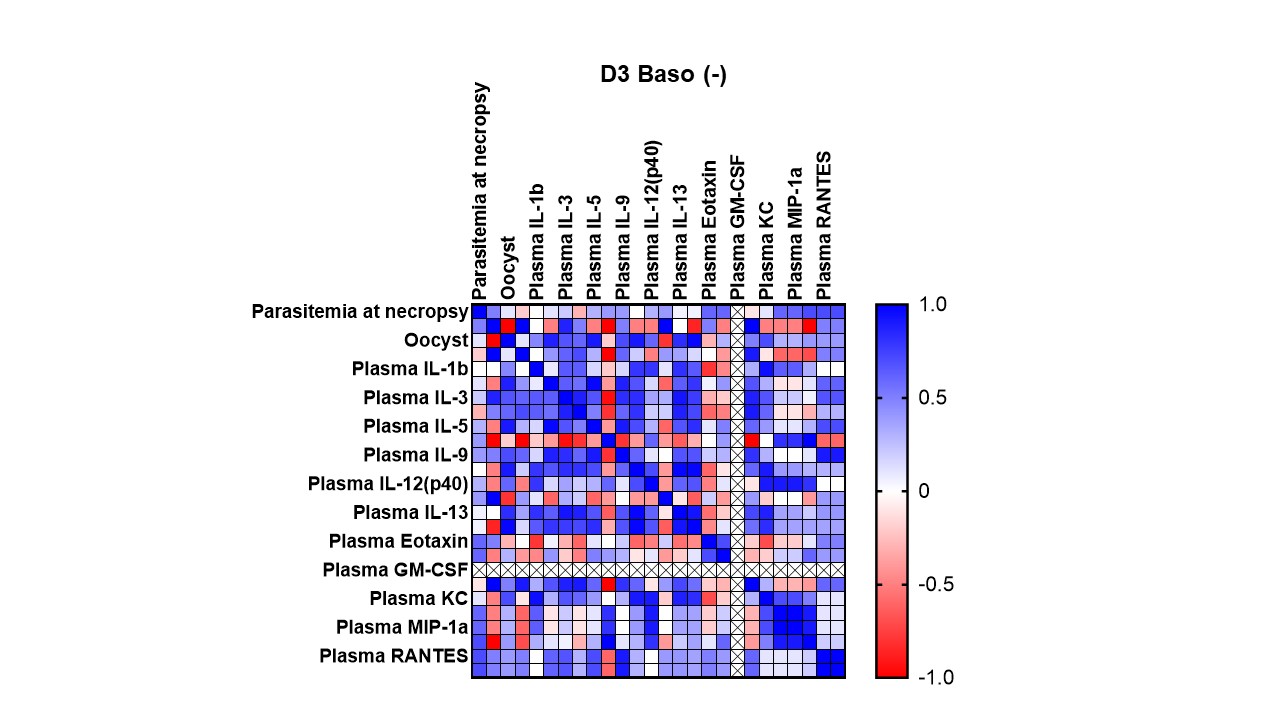

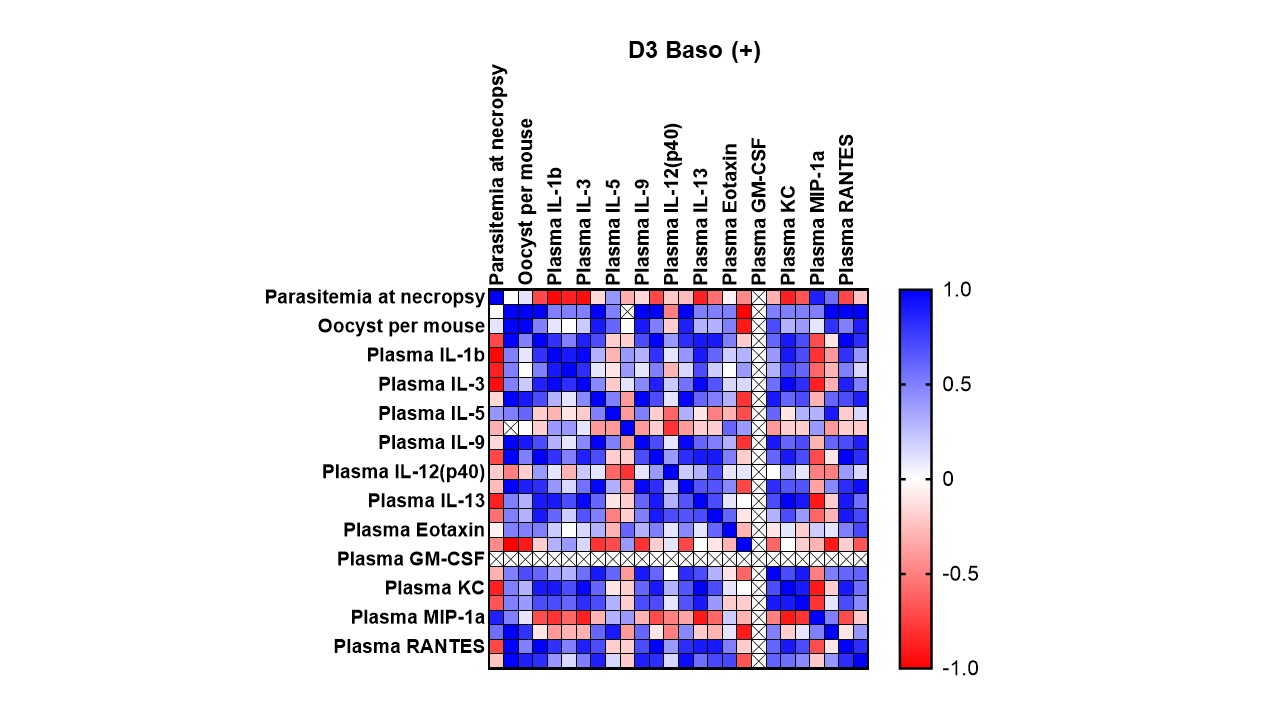


**A**

**B**


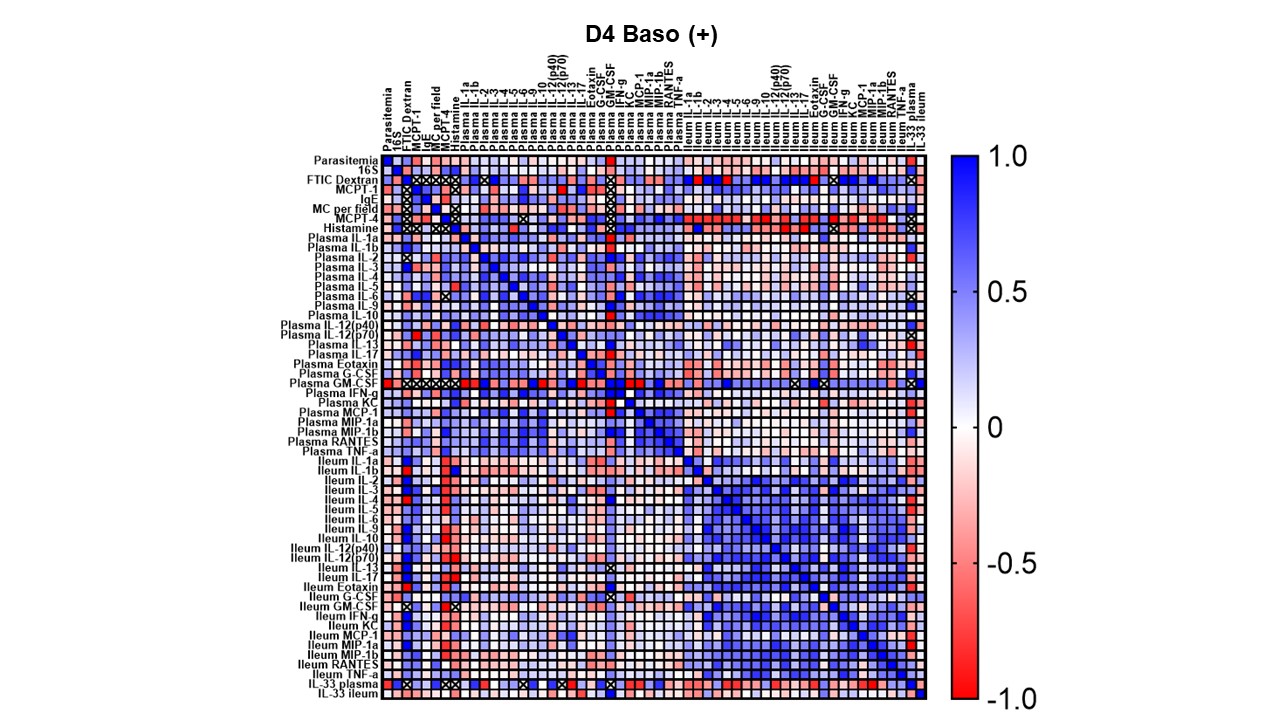

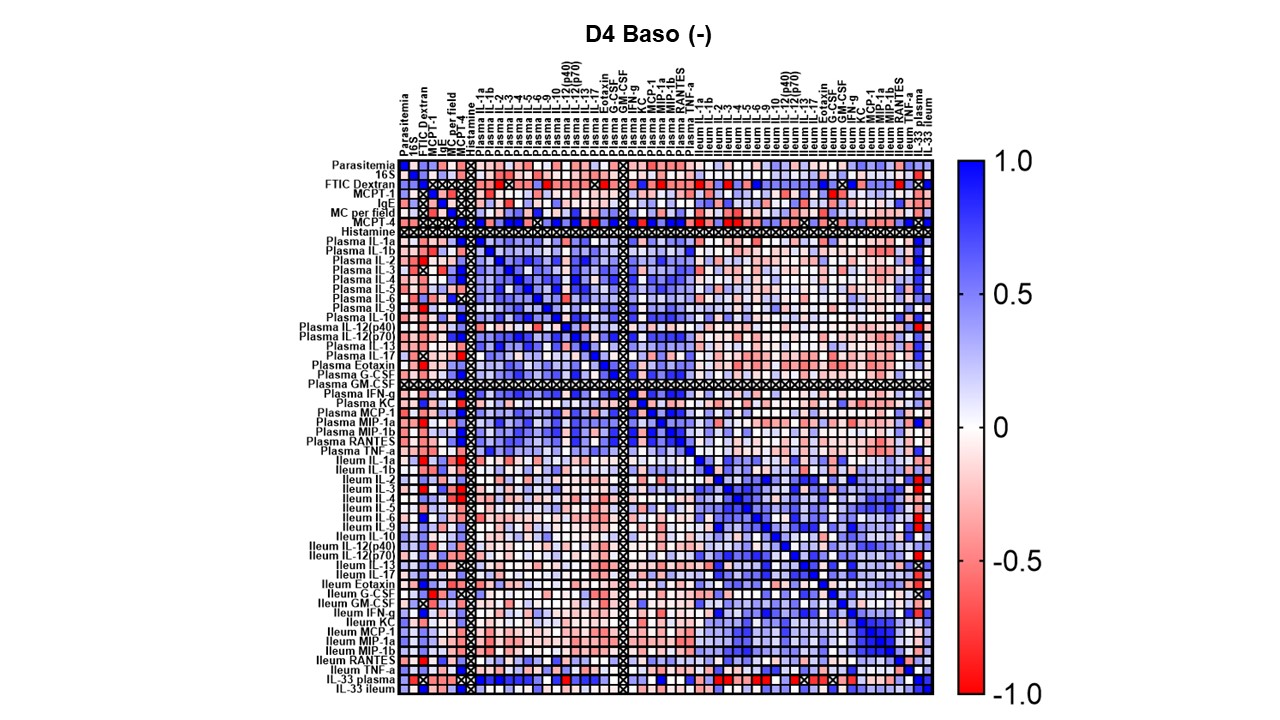


**D**

**C**


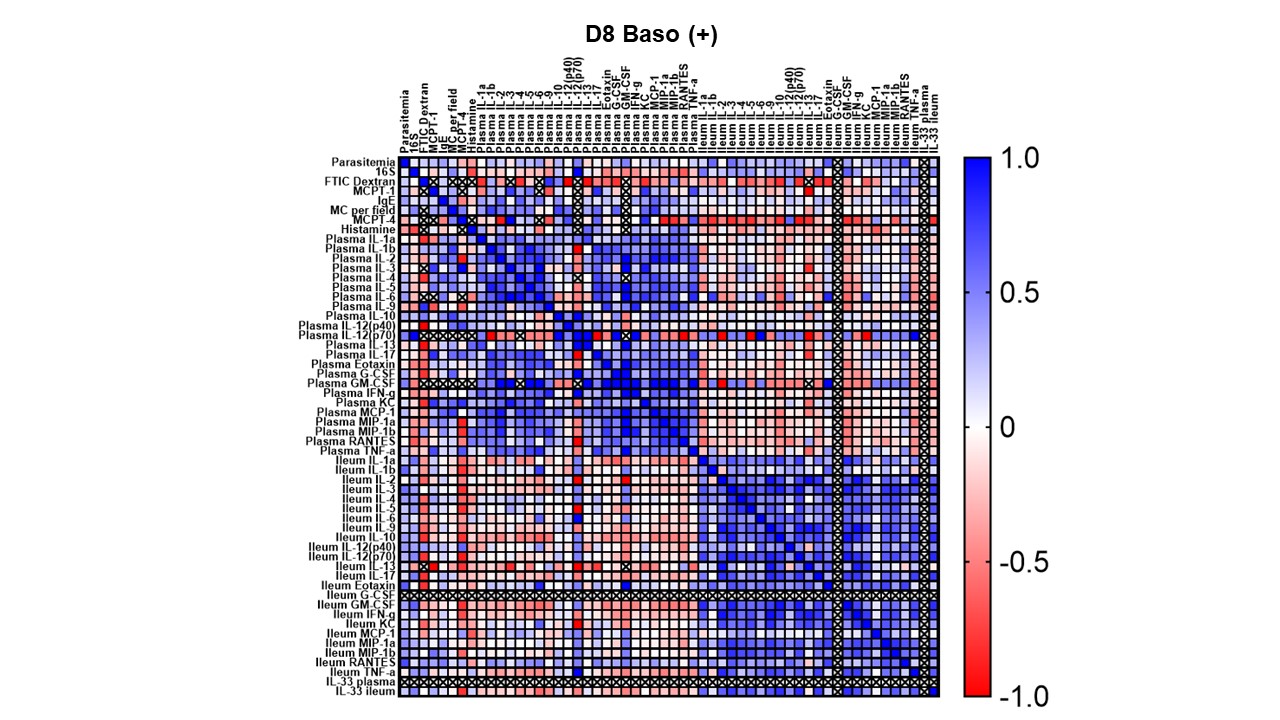

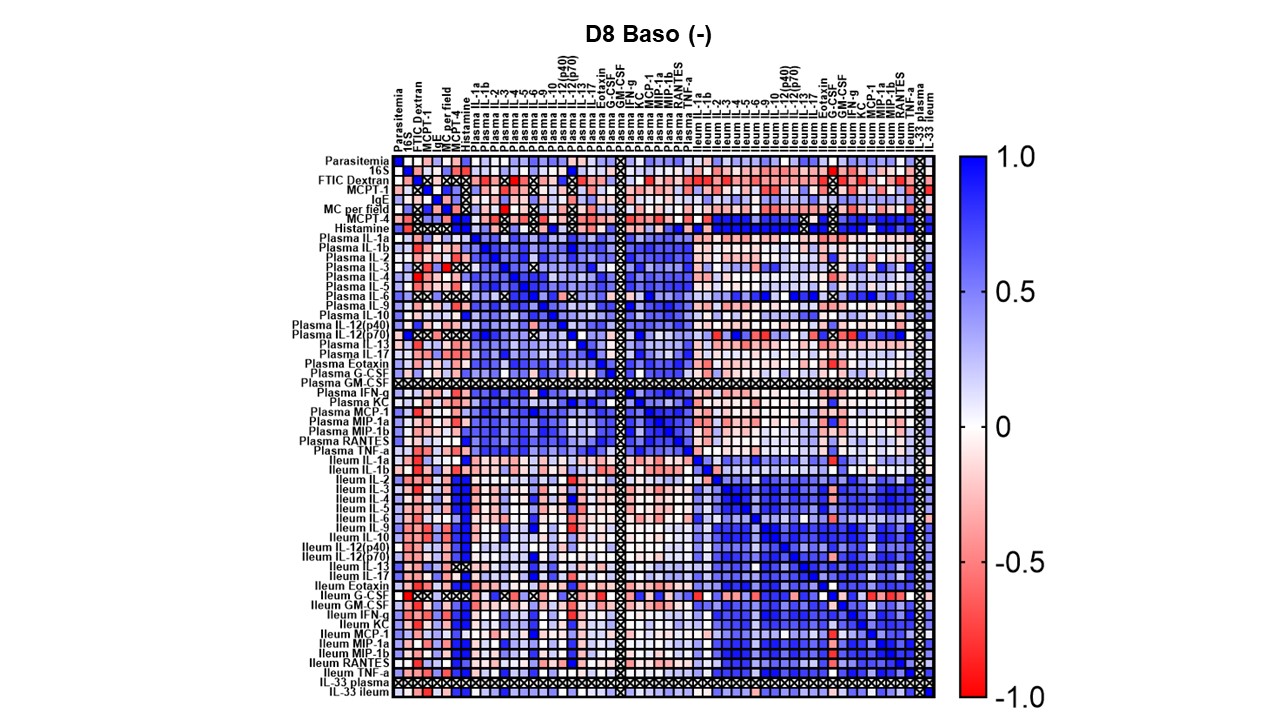

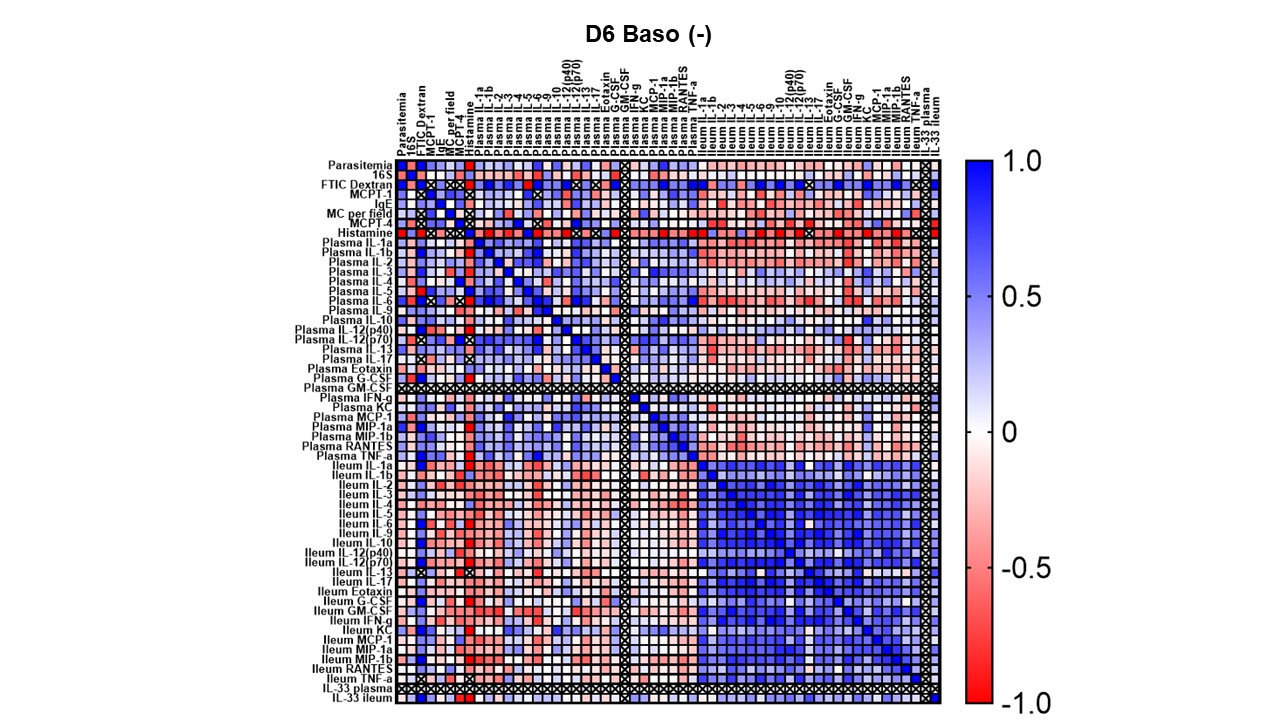

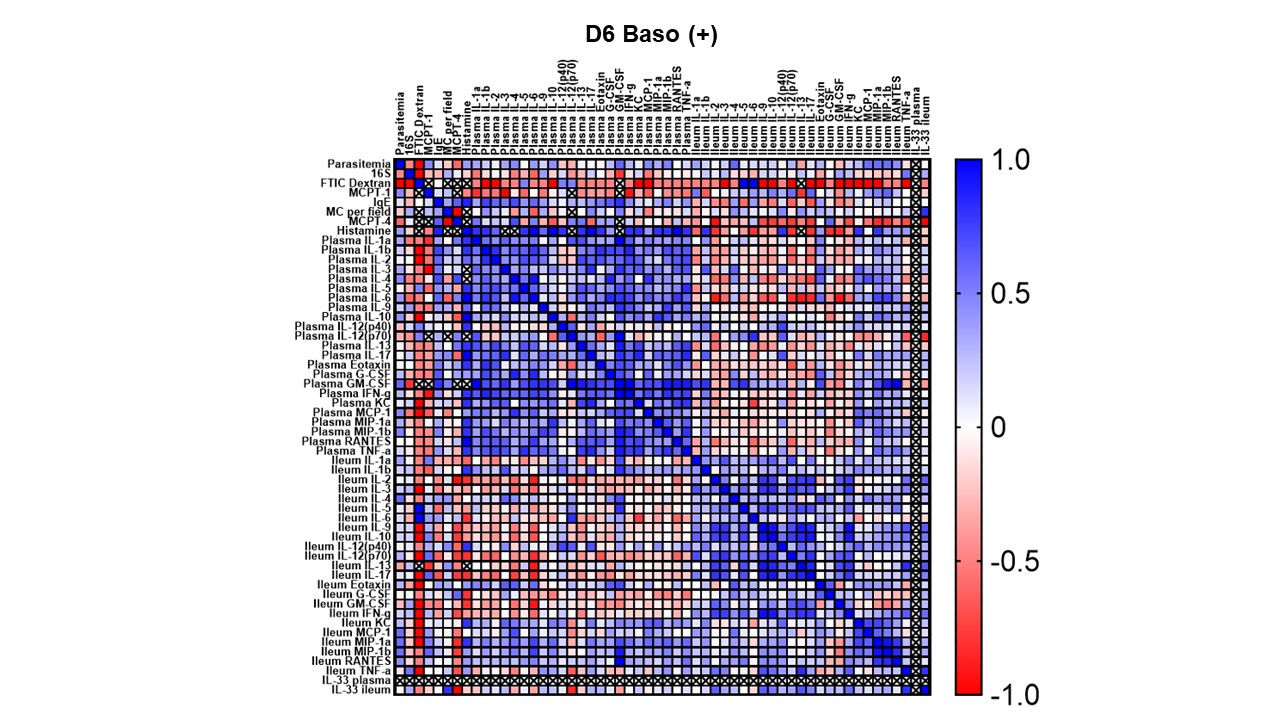


**H**

**G**

**F**

**E**

**Figure S7.** Correlation matrices used to build networks for day 3 (A, B), day 4 (C, D), day 6 (E, F) and day 8 (G, H) PI in basophil-depleted Baso(-) mice (n=62) and non-depleted Baso (+) mice (n=71). Correlations between parasitemia, gametocytemia, numbers of mosquito oocysts, bacterial 16S copies in blood, ileal MC numbers, levels of plasma and ileal cytokines and chemokines, plasma IgE, plasma Mcpt1, Mcpt4 and histamine were analyzed by Spearman test. Only significant correlations (P < 0.05) were used to build networks. Blue indicates at positive correlation between two factors, and red indicates a negative correlation between two factors. Strength of correlation is indicated by the intensity of color, with stronger correlations having deeper colors.
